# Arsenene-mediated multiple independently targeted reactive oxygen species burst for cancer therapy

**DOI:** 10.1038/s41467-021-24961-5

**Published:** 2021-08-06

**Authors:** Na Kong, Hanjie Zhang, Chan Feng, Chuang Liu, Yufen Xiao, Xingcai Zhang, Lin Mei, Jong Seung Kim, Wei Tao, Xiaoyuan Ji

**Affiliations:** 1 Center for Nanomedicine, Brigham and Women’s Hospital, Harvard Medical School, Boston, MA USA; 2 Academy of Medical Engineering and Translational Medicine, Medical College, Tianjin University, Tianjin, China; 3 School of Engineering and Applied Sciences, Harvard University, Cambridge, MA USA; 4 Tianjin Key Laboratory of Biomedical Materials, Key Laboratory of Biomaterials and Nanotechnology for Cancer Immunotherapy, Institute of Biomedical Engineering, Chinese Academy of Medical Sciences and Peking Union Medical College, Tianjin, China; 5 Department of Chemistry, Korea University, Seoul, Korea

**Keywords:** Cancer therapy, Biomaterials, Nanoscale materials

## Abstract

The modulation of intracellular reactive oxygen species (ROS) levels is crucial for cellular homeostasis and determination of cellular fate. A sublethal level of ROS sustains cell proliferation, differentiation and promotes tumor metastasis, while a drastic ROS burst directly induces apoptosis. Herein, surface-oxidized arsenene nanosheets (As/As_x_O_y_ NSs) with type II heterojunction are fabricated with efficient ·O_2_^−^ and ^1^O_2_ production and glutathione consumption through prolonging the lifetime of photo-excited electron-hole pairs. Moreover, the portion of As_x_O_y_ with oxygen vacancies not only catalyzes a Fenton-like reaction, generating ·OH and O_2_ from H_2_O_2_, but also inactivates main anti-oxidants to cut off the “retreat routes” of ROS. After polydopamine (PDA) and cancer cell membrane (M) coating, the engineered As/As_x_O_y_@PDA@M NSs serve as an intelligent theranostic platform with active tumor targeting and long-term blood circulation. Given its narrow-band-gap-enabled in vivo fluorescence imaging properties, As/As_x_O_y_@PDA@M NSs could be applied as an imaging-guided non-invasive and real-time nanomedicine for cancer therapy.

## Abstract (web summary)

Multifunctional materials with a number of effects are important for dealing with the complex environment in cancer therapy. Here, the authors report on surface-oxidized arsenene nanosheets coated with polydopamine and cancer cell membrane as a multi theranostic tumour targeting cancer therapy.

## Introduction

Reactive oxygen species (ROS) are a group of highly reactive small molecules generated by all aerobic organisms, which include hydroxyl radicals (·OH), superoxide (·O_2_^−^), and other nonradical members such as hydrogen peroxide (H_2_O_2_) and singlet oxygen (^1^O_2_)^1,2^. In particular, the redox balance of ROS between oxidizing and reducing species, through the cooperation of various enzymes (e.g., catalase, glutathione peroxidase and superoxide dismutase), is important in cell growth, proliferation, and signaling pathways^3–6^. However, the overproduction of ROS can cause oxidative stress, leading to cellular damage as well as the subsequent functional decline of organ systems^7^.

Although cancer cells are under higher oxidative stress because of the disrupted ROS homeostasis, they have adapted themselves to the overproduced ROS by activating antioxidant systems (e.g., the upregulation of glutathione, GSH)^4,8^. Ironically, they can utilize ROS to drive other events required for tumor development^1,3^. Hence, the modulation of intracellular ROS levels is crucial for tumor cellular homeostasis and determination of cellular fate, as different concentrations and durations of ROS stress can induce distinct biological responses. Therefore, only a powerful ROS burst to induce a high ROS level can cause effective cellular damage and apoptotic cell death.

Recently, a number of efforts have been devoted in chemodynamic therapy (CDT), photodynamic therapy (PDT), sonodynamic therapy (SDT) and other strategies to specifically increase the ROS level in cancer cells to mediate cell death^9–11^. However, to achieve a sufficient ROS burst, high concentrations of photo-/sono-sensitizers, high excitation energy, or harsh reaction environment are essential, which would in turn easily trigger adverse effects in normal tissues^7^. Therefore, elevating oxidative stress by multipartite generation of ROS preferentially in cancer cells with a low dose of sensitizers, lower excitation energy requirement, and simultaneous blockage of the ROS consumption pathway would be a logical and ideal therapeutic strategy. However, achieving the above-mentioned goal remains a critical bottleneck.

Two-dimensional (2D) nanomaterials with extraordinary physicochemical properties have attracted extensive attention for exciting applications in energy, catalysis, electronics, and biomedicines^12–17^. Driven especially by excellent optical and electronic properties and outstanding biocompatibility, mono-elemental 2D materials (e.g., borophene, phosphorene, antimonene, and germanene) have been developed as promising anticancer therapeutics: photosensitizers (PSs) for PDT and photothermal therapy (PTT), nano-carriers for drug delivery, as well as multi-modal imaging agents^18–25^. As an element in the same family of phosphorus, arsenene with its layered structure has recently been demonstrated to have characteristics similar to those of the buckled honeycomb structure of phosphorene and antimonene^26,27^. Owing to its suitably moderate band structure, high carrier mobility, as well as good optical properties, arsenene has been regarded as a promising candidate for photocatalysis, electrocatalysis, semiconducting devices, etc.^28,29^. Despite this substantial promise, not much efforts have been made to develop biomedical applications of arsenene^30,31^, especially its potential in cancer photonic therapies, such as PDT and PTT. Very recently, Wang and co-workers successfully synthesized arsenene nanosheet and applied it in an anticancer study, in which arsenene showed specific cytotoxicity against NB4 promyelocytic leukemia cells through affecting nuclear DNA replication, nucleotide excision repair, and pyrimidine metabolism pathways by downregulating the DNA polymerases (e.g., POLE, POLD1, POLD2, and POLD3)^32^. More importantly, considering that arsenic trioxide (As_2_O_3_) has now been approved by the U.S. Food and Drug Administration (FDA) as a frontline remedy for acute promyelocytic leukemia (APL)^33,34^, arsenene has been subjected to extensive pharmacological and clinical analysis, holds great promise for biomedical applications.

**Fig. 1:**
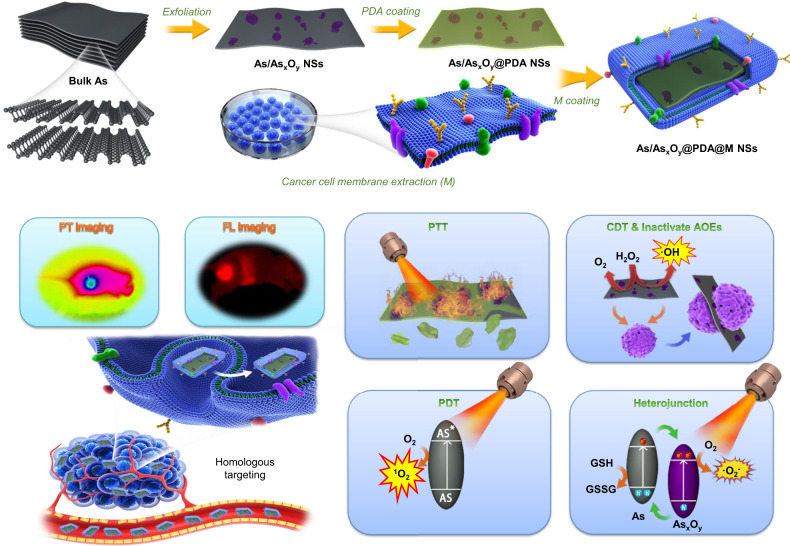
Schematic illustration of preparation and dual-modal imaging-guided cancer theranostics using As/As_*x*_O_*y*_@PDA@M NSs. Surface-oxidized arsenene nanosheets (As/As_*x*_O_*y*_ NSs) with type II heterojunction were fabricated by ball-grinding and liquid exfoliation. After polydopamine (PDA) and cancer cell membrane (M) coating, the engineered As/As_*x*_O_*y*_@PDA@M NSs serve as an intelligent theranostic platform with photothermal (PT) and fluorescence imagings guided photothermal therapy (PTT), photodynamic therapy (PDT), chemodynamic therapy (CDT), and inactivate antioxidant enzymes (AOEs).

Herein, we designed and fabricated a type II heterojunction photocatalyst based on arsenene, which is capable of multiple independently targeted ROS bursts but requiring a low-dose administration and less excitation energy. As shown in Fig. 1, by coupling ball-grinding with probe sonication-based liquid exfoliating processes, arsenene nanosheets with partial surface oxidation of As (As/As_*x*_O_*y*_ NSs) constructed type II heterojunction were synthesized. The photo-excited electrons in the conduction band (CB) of As can be transferred to the CB of As_*x*_O_*y*_ to catalyze the generation of ·O_2_^−^ from O_2_, while the holes in the valence band (VB) of As_*x*_O_*y*_ can be transferred to the VB of As to promote the oxidation of GSH. This type II heterojunction structure retards recombination of photogenerated electron–hole pairs of As/As_*x*_O_*y*_ NSs, which provides maximal power to catalyze the generation of ROS from O_2_ on the one hand, while blocking the consumption of ROS by GSH on the other hand. Additionally, the As in As/As_*x*_O_*y*_ NSs could generate ^1^O_2_ through energy conversion, and the As_*x*_O_*y*_ in As/As_*x*_O_*y*_ NSs could inactivate some main anti-oxidants and anti-oxidases containing sulfhydryl groups to cut off the “retreat routes” of ROS. Moreover, the oxygen vacancies in As/As_*x*_O_*y*_ NSs not only catalyze the disproportionate reaction of H_2_O_2_ via the high reduction ability of As^III^, but also generate O_2_ through H_2_O_2_ oxidation via the high oxidation capability of As^V^. Hence, the prepared As/As_*x*_O_*y*_ NSs can mediate synergetic tripartite ROS generation and block two main ROS consumption pathways, producing a dramatic ROS burst. In addition, its efficient photothermal conversion ability under 808 nm laser irradiation alongside its intrinsic fluorescence properties further enriched the biomedical applications of As/As_*x*_O_*y*_ NSs. With the aid of surface engineering using polydopamine (PDA) and cancer cell membrane (M) coating, the obtained As/As_*x*_O_*y*_@PDA@M NSs possess the advantages of a long blood circulation time, active cancer cell targeting, and tumor micro-environment (TME) responsive degradation, constituting excellent potential for in vivo fluorescence imaging-guided non-invasive and real-time cancer therapy.

In line of the revealed complete elimination of tumors without recurrence, our work presents arsenene-based nanomedicine with multiple independently targeted ROS bursts for effective cancer therapy. We also anticipate the appearance of such heterojunction photocatalysts will be attractive in other settings beyond biomedical applications.

## Results

### Fabrication and characterization of As/As_*x*_O_*y*_@PDA@M NSs

**Fig. 2:**
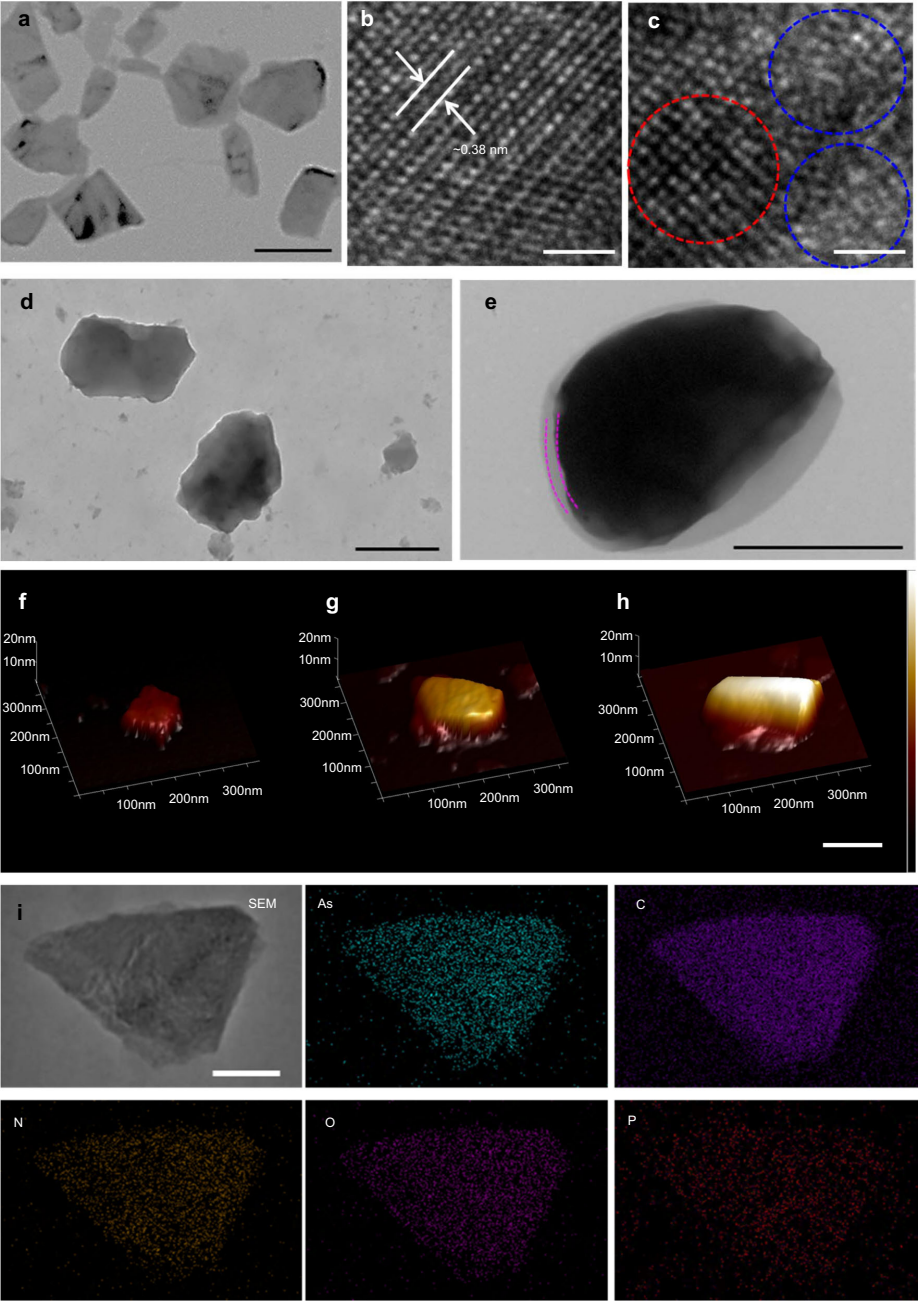
Morphology and composition characterization of ultrathin 2D As/As_*x*_O_*y*_@PDA@M NSs. **a** TEM images of As/As_*x*_O_*y*_ NSs, scale bar = 100 nm. **b** HRTEM images of As NSs, scale bar = 1 nm. **c** HRTEM images of As/As_*x*_O_*y*_ NSs (red circle: lattice fringes of As; blue circle: amorphism of As_*x*_O_*y*_), scale bar = 1 nm. **d** TEM images of As/As_*x*_O_*y*_@PDA NSs, scale bar = 100 nm. **e** TEM images of As/As_*x*_O_*y*_@PDA@M NSs, scale bar = 100 nm. **f**–**h** AFM images of As/As_x_O_*y*_ NSs, As/As_*x*_O_*y*_@PDA NSs, and As/As_*x*_O_y_@PDA@M NSs, respectively, scale bar = 100 nm. **i** SEM-EDS mapping of As/As_*x*_O_*y*_@PDA@M NSs: green (As), purple (C), orange (N), pink (O), and red (P), scale bar = 50 nm. For these morphology characterizations of fabricated As-based NSs, three times each experiment was repeated independently with similar results.

**Fig. 3:**
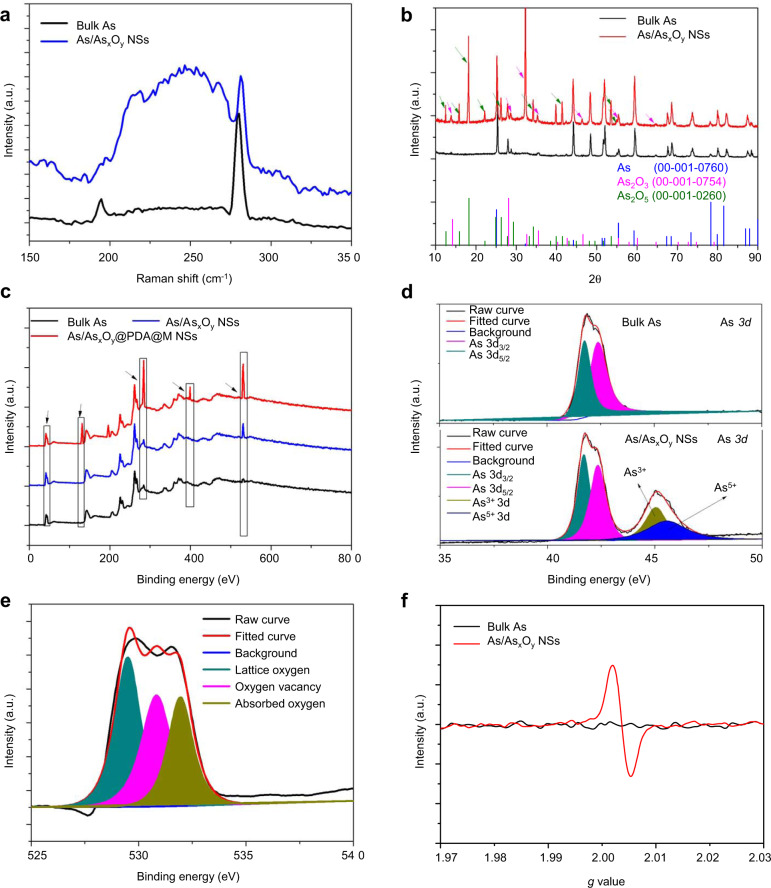
Chemical composition and structure characterization of ultrathin 2D As/As_*x*_O_*y*_@PDA@M NSs. **a** Raman shift spectra of bulk As and As/As_*x*_O_*y*_ NSs. **b** XRD spectra of bulk As and As/As_*x*_O_*y*_ NSs. **c** XPS spectra of bulk As, As/As_*x*_O_*y*_ NSs, and As/As_*x*_O_*y*_@PDA@M NSs. **d** HRXPS spectra of As 3*d* in bulk As and As/As_x_O_y_ NSs. **e** HRXPS spectra of O 1*s* in bulk As and As/As_*x*_O_*y*_ NSs. **f** ESR spectra of bulk As and As/As_*x*_O_*y*_ NSs. For these chemical characterizations of fabricated As based NSs, three times each experiment was repeated independently with similar results.

The synthesis of 2D ultrathin As/As_*x*_O_*y*_@PDA@M NSs is illustrated in Fig. 1, in which ball-grinding and liquid exfoliation were employed, and further PDA and cancer cell membrane coating were performed. In detail, layered As powder (Supplementary Fig. 1) with planar size about 5 μm immersed in N-methyl-2-pyrrolidone (NMP) solution was ground in a ball grinder, and As particles with planar size <1 μm were obtained. Next, the ground As powder dispersed in NMP was subjected to ultrasonic-assisted liquid exfoliation for 12 h. Surface-oxidized ultrathin As NSs (As/As_*x*_O_*y*_ NSs) were obtained with an average size of 93 nm and thickness of 3 nm, according to their TEM and AFM images, respectively (Fig. 2a, f, and Supplementary Fig. 2). To further confirm the successful fabrication of As/As_*x*_O_*y*_ NSs heterojunction, X-ray diffractometry (XRD), Raman detection, and X-ray photoelectron spectroscopy (XPS) were performed. Figure 3a showed the typical Raman spectra of the bulk As and the as-prepared As/As_*x*_O_*y*_ NSs with two peaks at 258 and 196 cm^−1^, which are consistent with the *A*_1*g*_ (out-of-plane vibration) and *E*_*g*_ (in-plane vibration) modes of arsenic, respectively^32^. In addition, a broadband of amorphous arsenic with weak intensity at 200–260 cm^−1^ was observed attributing to the formation of As_*x*_O_*y*_ on the surface of As NSs^35^, which indicated the amorphization of arsenene after exfoliation.

XRD detection of As/As_*x*_O_*y*_ NSs (Fig. 3b) further demonstrated successful heterojunction functionalization, in which three respective structures corresponding with As (JCPDS No. 00-001-0760), As_2_O_3_ (JCPDS No. 00-001-0754), and As_2_O_5_ (JCPDS No. 00-001-0260) were observed. In the XPS analysis (Fig. 3c), the peak intensity of O from the XPS spectrum of As/As_*x*_O_*y*_ NSs was much higher than that of bulk As powder, which should be ascribed to the result of surface oxidation. As shown in Fig. 3d, the typical As 3*d* high-resolution XPS spectra of metallic As were made up of two peaks at 42.3 and 41.6 eV, which were attributed to As 3*d*_3/2_ and As 3*d*_5/2_, respectively^36^. Notably, two other peaks at about 45 and 45.5 eV, in accordance with the As^3+^ (As^III^) and As^5+^ (As^V^) of As–O, respectively, were displayed in the As 3*d* high-resolution XPS spectra, which further confirmed the formation of As/As_*x*_O_*y*_ NSs. Additionally, the co-existence of the lattice fringes of As (Fig. 2b) and some amorphism of As_*x*_O_*y*_ (Fig. 2c) in As/As_*x*_O_*y*_ NSs also confirmed the successful fabrication of the heterojunction structure.

To improve the stability and photothermal conversion performance of As/As_*x*_O_*y*_ NSs, a PDA shell was introduced by self-polymerization of dopamine to form As/As_*x*_O_*y*_@PDA NSs. After PDA coating, the average size and thickness of As/As_*x*_O_*y*_@PDA NSs increased to 141 and 7 nm, respectively (Fig. 2d, g and Supplementary Fig. 2). To further achieve a good biocompatibility with initiative targeting ability, the As/As_*x*_O_*y*_@PDA NSs were further engineered to incorporate cancer cell membrane through an ultrasonic-assisted self-assembly strategy to form As/As_*x*_O_*y*_@PDA@M NSs. The successful deposition of cell membrane coating on the surface of As/As_*x*_O_*y*_@PDA NSs was clearly confirmed by TEM images of an obvious coating layer (Fig. 2e). Also, the planar size and thickness of As/As_*x*_O_*y*_@PDA@M NSs increased to ~156 nm (Supplementary Fig. 2) and 10 nm (Fig. 2h), respectively. Additionally, the membrane proteins from the cell membrane were well retained during this process (Supplementary Fig. 3). Moreover, XPS test indicated three new peaks of C, N, and P were present in As/As_*x*_O_*y*_@PDA@M NSs (Fig. 3c), which could be attributed to the PDA and M. Moreover, the elements As, C, N, O, and P were presented in the EDS mapping of As/As_*x*_O_*y*_@PDA@M NSs (Fig. 2i), and the zeta potential of differently functionalized As/As_*x*_O_*y*_ NSs (Supplementary Fig. 4) further demonstrated the successful surface coating of PDA and M shells.

### Characterization and analysis of mechanism underlying ROS burst ability

**Fig. 4:**
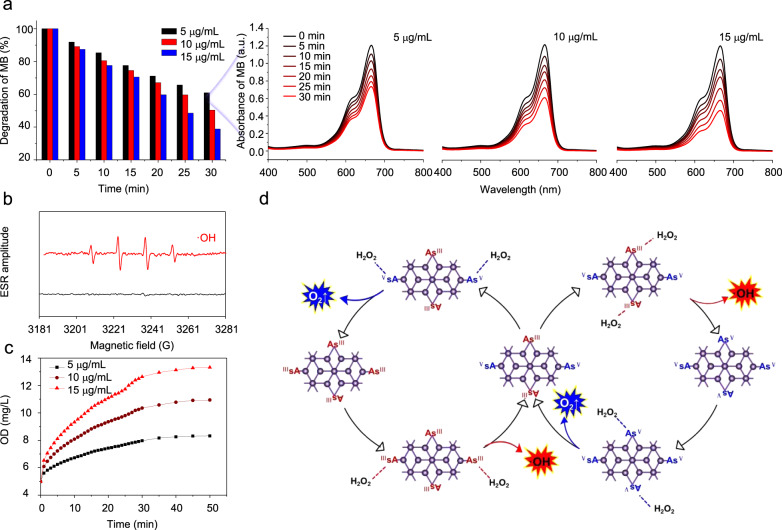
Fenton-like reaction-catalyzed ability of As/As_*x*_O_*y*_ NSs. **a** Degradation of MB caused by the generation of ·OH with different concentration of As/As_*x*_O_*y*_ NSs (5–15 μg/mL). **b** ESR spectra of ·OH generated by As/As_*x*_O_*y*_ NSs. **c** O_2_ generation by As/As_*x*_O_*y*_ NSs at different concentrations (5–15 μg/mL). **d** The mechanism of As/As_*x*_O_*y*_ NSs for generation of ·OH and O_2_. As^III^ with high reducibility catalyzes the disproportionate reaction of H_2_O_2_ to generate ·OH, and As^V^ with high oxidizability catalyzes the oxidation of H_2_O_2_ to generate O_2_ and realizes regeneration of As^III^. Three times each experiment was repeated independently with similar results.

The deconvoluted As 3*d* spectra of As/As_*x*_O_*y*_ NSs presented two peaks at 45 and 45.5 eV, which were in line with the binding energy of As^3+^ (As_2_O_3_) and As^5+^ (As_2_O_5_), respectively. In addition, compared with bulk As, the binding energies of 3*d* As in As/As_*x*_O_*y*_ NSs was lower, which might be ascribed to the introduction of oxygen vacancy into As/As_*x*_O_*y*_ NSs. To confirm the formation of oxygen vacancy on the surface of As/As_*x*_O_*y*_ NSs, HRXPS and EPR measurements were employed. As shown in the O 1*s* scans (Fig. 3e), there were three O species attributed to lattice oxygen (e.g., As_2_O_3_ and As_2_O_5_) in the surface of As/As_*x*_O_*y*_ NSs (529.5 eV), oxygen vacancies (530.5 eV), and absorbed oxygen on the surface of samples (532.2 eV). To analyze the trapped electrons in As samples, ESR spectroscopy was carried out (Fig. 3f). Bulk As showed weak ESR signals, indicating a very low percentage of defects. By contrast, the as-exfoliated As/As_*x*_O_*y*_ NSs exhibited a symmetrical pair of peaks, attributable to the trapped unpaired electrons from the oxygen vacancies that adsorbed oxygen species from air. As is well-known, numerous metallic oxides with oxygen-deficient structures exhibit excellent behaviors in the fields of catalysis and energy conversion^37,38^. Herein, methylene blue (MB) was employed to study ·OH generation ability through Fenton-like reactions between As/As_*x*_O_*y*_ NSs and H_2_O_2_^39^. Figure 4a presented the degradation of MB under different concentrations of As/As_*x*_O_*y*_ NSs in the presence of H_2_O_2_. Considerable MB degradation was observed with the increased concentrations of As/As_*x*_O_*y*_ NSs. Moreover, ESR measurements further confirmed the generation of ·OH via Fenton-like reactions catalyzed by the As/As_*x*_O_*y*_ NSs with plenty of oxygen vacancies (Fig. 4b). Obvious O_2_ generation was also observed (Fig. 4c), which could not only relieve the hypoxic microenvironment in the tumor, but also enhance the efficiency of PDT mediated by As/As_*x*_O_*y*_ NSs. The mechanism of As/As_*x*_O_*y*_ NSs-mediated Fenton-like reactions for the generation of ·OH and O_2_ was explained in Fig. 4d. In detail, after exfoliation-assisted surface oxidation, As_2_O_3_ and As_2_O_5_ were found on the surface of As/As_*x*_O_*y*_ NSs. Abundant oxygen vacancies were detected on the surface of As/As_*x*_O_*y*_ NSs, which could not only catalyze the disproportionate reaction of H_2_O_2_ via the high reduction ability of As^III^, but also generate O_2_ through H_2_O_2_ oxidation by the high oxidation ability of As^V^. As a consequence, the Fenton-like reaction enables the As/As_*x*_O_*y*_ NSs to produce ·OH (one of most toxic ROS), and to relieve the hypoxic tumor microenvironment by self-generation of O_2_, which further served as a substrate for enhancing PDT.

**Fig. 5:**
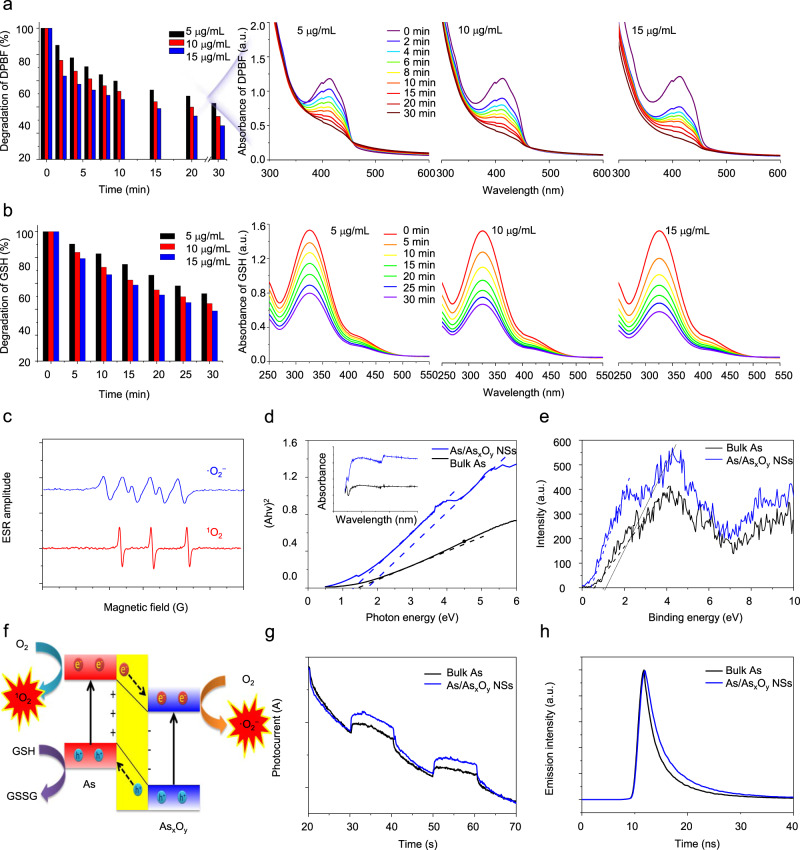
Photocatalytic performance of As/As_*x*_O_*y*_ NSs. The degradation of **a** DPBF and **b** GSH caused by As/As_*x*_O_*y*_ NSs under 660 nm laser irradiation at 0.3 W/cm^2^. **c** ESR spectra of ·O_2_^-^ and ^1^O_2_ generated by As/As_*x*_O_*y*_ NSs under 660 nm laser irradiation at 0.3 W/cm^2^. **d** UV–Vis absorbance spectra and calculated band gap of bulk As and As/As_*x*_O_*y*_ NSs. **e** Valence band of bulk As and As/As_*x*_O_*y*_ NSs calculated from XPS spectra. **f** Mechanism of type II heterojunction based on As/As_*x*_O_*y*_ NSs for ·O_2_^−^ and ^1^O_2_ generation and GSH oxidation. **g** Photocurrent curve of bulk As and As/As_*x*_O_*y*_ NSs under 660 nm laser irradiation. For the photocurrent analysis, three electrodes were used: glassy-carbon (working), silver–silver chloride (reference), and platinum (counter) electrodes in a sodium phosphate buffer (100 mM, pH 7.0). **h** Time-resolved fluorescence spectra of As and As/As_*x*_O_*y*_ NSs. Three times each experiment was repeated independently with similar results.

Next, the performance of As/As_*x*_O_*y*_ NSs as an efficient photosensitizer for inducing ROS burst was investigated in depth. As shown in Fig. 5a, an obvious degradation of 1,3-diphenylisobenzofuran (DPBF) was observed after the treatment of As/As_*x*_O_*y*_ NSs at a low concentration (5–15 μg/mL) plus 660 nm laser irradiation even under a low power density (0.3 W cm^−2^), which demonstrated the ROS generation from O_2_ under the strong catalyzing effect of As/As_*x*_O_*y*_ NSs. More interestingly, GSH oxidation was also noted in case of As/As_*x*_O_*y*_ NSs plus 660 nm laser irradiation (Fig. 5b), which indicated that As/As_*x*_O_*y*_ NSs could catalyze both the reduction of O_2_ and oxidation of GSH, inducing ROS aggregation directly and indirectly, respectively.

For a detailed analysis of the type of ROS, ESR was employed by using 5,5-dimethyl-1-pyrrolineN-oxide as a spin trapping agent. Interestingly, two types of ROS (·O_2_^−^ and ^1^O_2)_ were detected (Fig. 5c). The mechanism for the generation of ^1^O_2_ was similar to that of black phosphorus (BP) NSs and most organic small molecule photosensitizers, which should be ascribed to the absorbed light energy transferred to O_2_ and mediated by As NSs. To further confirm the mechanism of GSH depletion and ·O_2_^−^ generation of As/As_x_O_y_ NSs under 660 nm laser irradiation, XPS spectra and solid UV–vis–NIR absorbance spectra of the As/As_*x*_O_*y*_ NSs were measured to reveal their electronic band structure. Figure 5d demonstrates that the prepared As/As_*x*_O_*y*_ NSs have a much stronger light-absorbance ability than bulk As. As shown in Fig. 5d, two curves resulting from the heterogeneous structure of As/As_*x*_O_*y*_ NSs were observed, and the band gaps (*E*_g_) of As and As_*x*_O_*y*_ in As/As_*x*_O_*y*_ NSs were calculated to be 1.4 and 1.7 eV, respectively. Similarly, from the XPS spectra, the VB values of As and As_*x*_O_*y*_ of As/As_*x*_O_*y*_ NSs were calculated to be 0.4 and 1.2 eV, respectively (Fig. 5e). Next, from the difference between Eg and VB, the CB values of As and As_*x*_O_*y*_ were determined to be −1.0 and −0.5 eV, respectively. The energy bands and electron transfer in As/As_*x*_O_*y*_ NSs are presented in Fig. 5f. To be specific, when exposed to 660 nm laser, the As and As_*x*_O_*y*_ of As/As_*x*_O_*y*_ NSs were simultaneously excited. The electrons in the CB of As in the developed type II As/As_*x*_O_*y*_ NSs heterojunction preferably transfer to the CB of As_*x*_O_*y*_. In the meantime, the holes in the VB of As_*x*_O_*y*_ preferred to transfer to the VB of As, contributing to the thoroughly separate photo-excited charges into the CB and VB of two different photosensitizers, which fundamentally avoided electron–hole pair recombination. In addition, the *E*^0^ of O_2_/·O_2_^−^ (−0.16 eV) and the *E*^0^ of GSH/GSSG (0.3 eV) were lower than that of the CB of As_*x*_O_*y*_ and the VB of As, respectively. Therefore, from a thermodynamic point of view, it became possible to transfer electrons from the CB of As_*x*_O_*y*_ to O_2_ for the generation of·O_2_^−^ and capture of electrons by VB of As to produce GSSG from GSH. Moreover, the photochemical properties of the As/As_*x*_O_*y*_ NSs-based type II heterojunction were further investigated. As shown in Fig. 5g, the photocurrent response versus time of As/As_*x*_O_*y*_ NSs was higher than that of As NSs. The photocurrent response of As/As_*x*_O_*y*_ NSs was reversible and stable under the increased current and was quenched reproducibly by on/off irradiation. The high photocurrent of As/As_*x*_O_*y*_ NSs was responsible for the super-photocatalytic activity. This indicated that the electrons excited by 660 nm laser were transferred from the GSH to the electrode through the As/As_*x*_O_*y*_ NSs. To confirm the suppressed recombination of electron–hole pairs in As/As_*x*_O_*y*_ NSs, time-resolved fluorescence decay analysis was further performed. As shown in Fig. 5h, the As/As_*x*_O_*y*_ NSs possessed a longer fluorescence lifetime than As NSs, indicating that the incorporation of As and As_*x*_O_*y*_ with the formed type II heterojunction increased the photoelectron lifetime of As/As_*x*_O_*y*_ NSs. This result further confirmed that As/As_*x*_O_*y*_ NSs-based type II heterojunction provides a much more efficient light energy transfer and could also mediate a highly efficient ROS burst directly and indirectly.

### Photothermal conversion and TME-responsive biodegradation

**Fig. 6:**
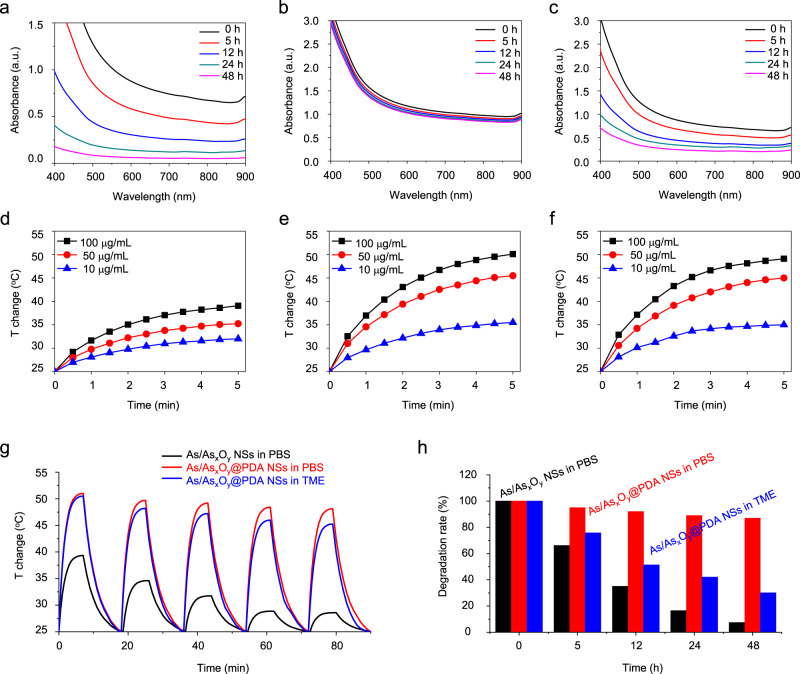
Photothermal conversion and stability of As/As_*x*_O_*y*_@PDA NSs. UV–Vis absorbance spectra of **a** As/As_*x*_O_*y*_ NSs and **b** As/As_*x*_O_*y*_@PDA NSs in PBS solution for different time intervals. **c** UV–Vis absorbance spectra of As/As_*x*_O_*y*_@PDA NSs in TME-mimicking solution for different time periods. Photothermal conversion curves of **d** As/As_*x*_O_*y*_ NSs and **e** As/As_*x*_O_*y*_@PDA NSs in PBS solutions. **f** Photothermal conversion curves of As/As_*x*_O_*y*_@PDA NSs in TME-mimicking solution. **g** Photothermal conversion stability of As/As_*x*_O_*y*_ NSs and As/As_*x*_O_*y*_@PDA NSs in PBS and in TME-mimicking solutions, respectively. **h** Degradation performance of As/As_*x*_O_*y*_ NSs and As/As_*x*_O_*y*_@PDA NSs in PBS and TME-mimicking solutions, respectively. Three times each experiment was repeated independently with similar results.

After confirming the excellent ability of As/As_*x*_O_*y*_ NSs for mediating ROS burst in vitro, the biodegradability of As/As_*x*_O_*y*_ NSs and As/As_*x*_O_*y*_@PDA NSs dispersed in PBS and TME mimetic solution were examined. As presented in Fig. 6a, the As/As_*x*_O_*y*_ NSs showed fast degradation in PBS solution, which is fatal for storage and blood circulation of nanoagents. After PDA coating, the As/As_*x*_O_*y*_@PDA NSs demonstrated excellent stability under normal physiological conditions. Vis–NIR absorbance spectra of As/As_*x*_O_*y*_@PDA NSs incubated in PBS indicated good stability (Fig. 6b), which bodes well for storage and blood circulation. The biodegradability of As/As_*x*_O_*y*_@PDA NSs under simulated TME condition was further measured. As shown in Fig. 6c, TME-responsive biodegradability was also observed.

The potential of NIR-induced hyperthermia of 2D nanomaterials is regarded as a promising cancer therapy strategy. To assess photothermal conversion performance, the As/As_*x*_O_*y*_ NSs and As/As_*x*_O_*y*_@PDA NSs at different concentrations (Supplementary Fig. 5) were exposed to an 808 nm laser (2 W cm^−2^). As presented in Fig. 6d, definite photothermal conversion ability of As/As_*x*_O_*y*_ NSs was observed, with the greatest temperature change (Δ*T*_max_) of 14 °C after laser irradiation (5 min, 2 W cm^−2^). Nevertheless, the photothermal conversion efficiency of As/As_*x*_O_*y*_ NSs was much lower than that of other recently reported 2D nanomaterials^24,40,41^. In addition, the As/As_*x*_O_*y*_ NSs exhibited very poor photothermal stability (Fig. 6g), which may be because the high-temperature accelerated degradation. As reported previously, the PDA shell not only improved the stability of As/As_*x*_O_*y*_ NSs in normal physiological environment, but also exhibited a robust photothermal conversion performance^42^. Hence, the As/As_*x*_O_*y*_@PDA NSs possess excellent photothermal conversion efficiency, with the greatest temperature change (Δ*T*_max_) of 24 °C after NIR laser irradiation (5 min, 2 W cm^−2^) (Fig. 6e). Moreover, the photothermal stability of As/As_*x*_O_*y*_@PDA NSs was further examined by exposing the As/As_*x*_O_*y*_@PDA NSs solution to the laser for five on/off irradiation cycles. As displayed in Fig. 6g, negligible changes in photothermal conversion ability were observed, clearly suggesting the high photothermal stability of As/As_*x*_O_*y*_@PDA NSs. Further, photothermal conversion performance of As/As_*x*_O_*y*_@PDA NSs in a TME-mimicking solution was also assessed (Fig. 6f). The photothermal conversion ability of As/As_*x*_O_*y*_@PDA NSs was observed similarly to that of in PBS solution for the first cycle, and a slight decrease of which was noticed after five cycles, which also demonstrated the TME-responsive biodegradability of As/As_*x*_O_*y*_@PDA NSs. As shown in Supplementary Fig. 6, the photothermal conversion ability of As/As_*x*_O_*y*_@PDA@M NSs was not changed after membrane coating.

### Active cancer cell targeting and fluorescence imaging in vitro

**Fig. 7:**
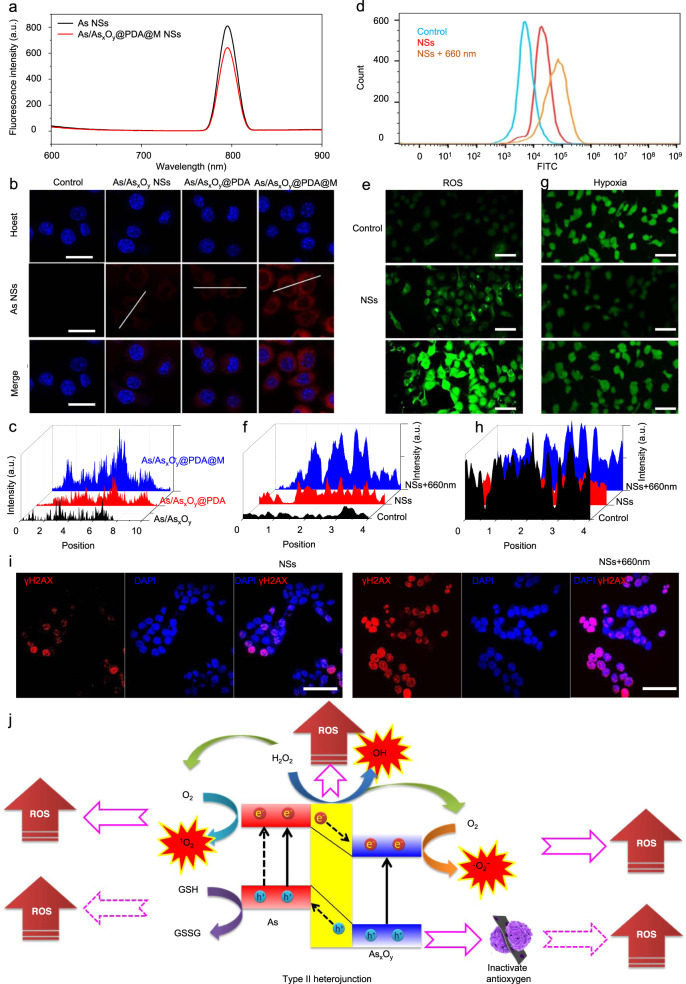
Intracellular uptake and ROS generation. **a** Fluorescence spectrum of As NSs and As/As_*x*_O_*y*_@PDA@M NSs with the excitation wavelength at 500 nm and emission wavelength at 795 nm. **b** Intracellular uptake of NSs in various formations after incubation for 4 h characterized by LSCM with the excitation wavelength at 500 nm and emission wavelength at 795 nm. Scale bars = 50 μm. **c** Fluorescence quantitative analysis of the intracellular uptake of NSs. **d** Intracellular ROS generation detected by FCM. **e** and **g** Intracellular ROS and O_2_ generation detected by CLSM. Scale bars = 100 μm. **f** and **h** Fluorescence quantitative analysis of intracellular ROS and O_2_ generation. **i** Representative confocal microscopy images of the MCF-7 cells (scale bars, 50 μm) after different treatments. The nuclei were stained by DAPI (blue), and the γH2AX foci per nucleus were stained by anti-γH2AX antibody (red). **j** Mechanism of As/As_*x*_O_*y*_@PDA@M NSs for modulating ROS burst. The engineered As/As_*x*_O_*y*_@PDA@M NSs serve as an intelligent theranostic platform-mediated ROS burst through five pathways. For the intracellular uptake, ROS and O_2_ generation, and DNA damage of developed NSs, three times each experiment was repeated independently with similar results.

Although nanoagents with size of 20–200 nm have the advantage of passive accumulation through the enhanced permeability and retention (EPR) effect in tumors, creating nanoagents with active targeting ability through cancer cell membrane engineering is still one of the most important goals of nanomedicine^43^. As shown in Fig. 7a, due to the narrow-band-gap of As NSs, As/As_*x*_O_*y*_@PDA@M NSs exhibited obvious photoluminescence (PL) property. It is well known that the PL signals of semiconductor materials result from the recombination of photo-induced charge carriers. As mentioned before, the photo-excited process of As/As_*x*_O_*y*_@PDA@M NSs occurred upon laser irradiation, in which the electrons from the VB are promoted to the CB with simultaneous generation of holes in the VB. However, the excited electrons in the CB easily come back to the VB via a certain course to recombine with the holes. During the recombination process of photo-induced charge carriers, a certain amount of chemical energy can be released, which would further transform possibly to light energy. As exhibited in Fig. 7a, As NSs showed a relatively strong PL signal (excitation: 500 nm; emission: 795 nm), which also demonstrated serious recombination of photo-induced charge carriers. The decreased PL signal of As/As_*x*_O_*y*_@PDA@M NSs further confirmed the retard recombination of photo-induced charge carriers through type II heterojunction. Additionally, although the type II heterojunction retarded the recombination to some extent, there were still a certain amount of photo-induced charge carriers recombined in VB, which was the main reason for the PL phenomenon of As/As_*x*_O_*y*_@PDA@M NSs both in vitro and in vivo. To investigate the active targeting and accumulation ability of As/As_*x*_O_*y*_@PDA@M NSs in tumor cells, the cellular uptake efficiency on MCF-7 cells was measured. After 2 h incubation, as shown in the cell fluorescence images (Fig. 7b), cells treated with As/As_*x*_O_*y*_@PDA@M NSs presented a much higher fluorescence intensity in their cytoplasm compared with that of As/As_*x*_O_*y*_ NSs-treated or As/As_*x*_O_*y*_@PDA NSs-treated cells. Quantitative analysis of red fluorescence intensity in cellular cytoplasm provided further evidence for the effective active targeting and accumulation ability of As/As_*x*_O_*y*_@PDA@M NSs in tumor cells (Fig. 7c).

### ROS burst in vitro

Next, the properties of As/As_*x*_O_*y*_@PDA@M NSs for inducing ROS burst were investigated on a cellular level by employing 2,7-dichlorofluorescin diacetate (DCFH-DA) as the intracellular ROS detection probe, which could be oxidized by ROS to emit green fluorescence. As exhibited in Fig. 7d–f and Supplementary Fig. 7, cancer cells treated with As/As_*x*_O_*y*_@PDA@M NSs showed an obvious ROS production compared with that of the control group, which could be assigned to the following two causes. First, as mentioned earlier, the As/As_*x*_O_*y*_ NSs possessed a Fenton-like reactions-based catalyzed ability, which could disproportionately catalyze the reaction of H_2_O_2_ to generate ·OH and O_2_. Second, as previously reported, As_2_O_3_ has a high affinity to mercapto groups^44^, which could bind to anti-oxidants and anti-oxidases containing sulfhydryl groups in cells. Therefore, As_2_O_3_ would reduce cell clearance and antagonism to oxidation, breaking the dynamic balance of intracellular anti-oxidation, and contributing to increased intracellular ROS level. Moreover, the strongest green fluorescence signal was observed in cells treated with As/As_*x*_O_*y*_@PDA@M NSs plus 660 nm laser irradiation treatment. Additionally, a hypoxic probe was also employed to evaluate the TEM after different treatments. After treatment with As/As_*x*_O_*y*_@PDA@M NSs, a weak green fluorescence indicating the production of some O_2_ confirmed the O_2_-elevating ability of As/As_*x*_O_*y*_@PDA@M NSs via Fenton-like reactions (Fig. 7g and h). As a result of the photodynamic effect of As/As_*x*_O_*y*_@PDA@M NSs, the concentration of O_2_ was decreased upon the 660 nm laser irradiation (Fig. 7g and h). It is well known that high ROS content will induce high levels of irreparable DNA damage in cancer cells. To visually observe the irreparable DNA damage levels in MCF-7 cells after different treatments, immunofluorescent staining assay was performed to detect the irreparable DNA damage in the nucleus using γ-H2AX to stain DNA damage sites. As shown in Fig. 7i and Supplementary Fig. 8, the treatment of As/As_*x*_O_*y*_@PDA@M NSs induced obvious irreparable DNA damage in MCF-7 cells. Moreover, the treatment of As/As_*x*_O_*y*_@PDA@M NSs coupling with 660 nm laser irradiation induced remarkably high levels of irreparable DNA damage in cancer cells. These findings further confirmed the highly efficient photodynamic effect mediated by the type II heterojunction-based As/As_*x*_O_*y*_@PDA@M NSs to catalyze ·O_2_^−^ and ^1^O_2_ generation on the one hand, and to deplete GSH on the other hand, leading to the desired intracellular ROS burst (Fig. 7j).

### Antitumor strategy in vitro

The phototherapy, especially PDT, has been clinically applied or under clinical trials to treat lung cancers and breast cancers^45^. For instance, during 1994–1998, Porfimer sodium, an organic photosensitizer, was granted regulatory approval for PDT towards non-small-cell lung cancer among serval countries (e.g., Japan, USA, Canada, Germany, and UK). Thereafter, some other photosensitizers (e.g., Talaporfin) were also approved for PDT of lung cancer. As for breast cancer, treatment of primary breast cancer using PDT has been carried out with results pending (e.g., ClinicalTrials.gov Identifier: NCT02872064). Moreover, although the therapeutic efficacy of light-assisted PDT and PTT might be hindered by the penetration depth of light, using a different light-delivery approach (e.g., light fiber) could be performed on lung and breast tumors. For instance, laser interstitial thermal therapy (LITT), generally involving placement of laser fibers into tumors, has been explored for the treatment of benign breast tumor (ClinicalTrials.gov Identifier: NCT00807924). Considering the clinical application of phototherapy for the treatment of lung and breast tumors, we, therefore, use A549 (lung cancer cells) and MCF-7 (breast cancer cells) cell lines to demonstrate the practicality of our developed As/As_*x*_O_*y*_@PDA@M NSs.

**Fig. 8:**
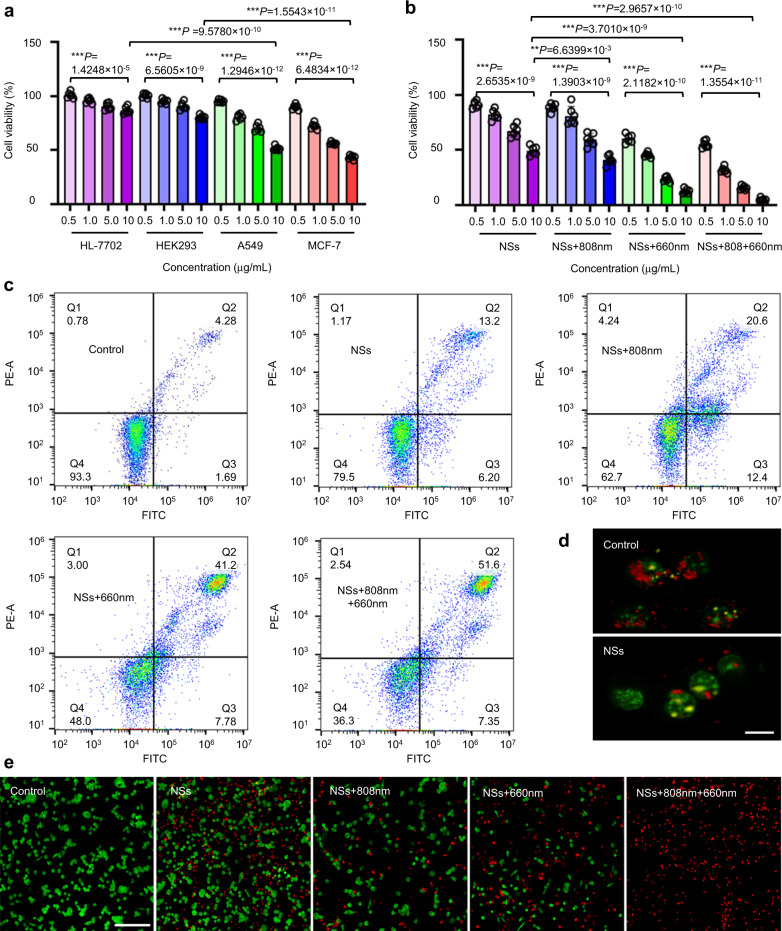
Biocompatibility and cytotoxicity of As/As_*x*_O_*y*_@PDA@M NSs. **a** Relative viability of HL-7702, HEK293, A549, and MCF-7 cells after incubation with As/As_*x*_O_*y*_ NSs for 24 h. **b** Relative viability of MCF-7 cells after incubation with As/As_*x*_O_*y*_@PDA@M NSs under different treatments for 24 h. The power of 660 nm laser and 808 nm laser were 0.3 and 1.0 W cm^−2^, respectively. The exposure time was 10 min. Error bars = standard deviation (*n* = 6), *n* = 6 biologically independent cells. Data are presented as mean values ± SEM. Two-sided ANOVAs were performed for all other comparisons. No adjustments were made for multiple comparisons. **c** FCM images of MCF-7 cells after incubation with As/As_*x*_O_*y*_@PDA@M NSs under different treatments for 12 h. **d** Fluorescence microscopy images of mitochondrial membrane potential change detected by JC-1 staining (Red: hyperpolarization; Green: depolarization) (scale bars = 10 μm). **e** Florescent images of MCF-7 cells stained with calcein AM (Green: live cells) and PI (Red: dead cells) (scale bars = 200 μm). For the mitochondrial membrane potential change and live/dead cells staining, three times each experiment was repeated independently with similar results.

Next, the cytotoxicity of prepared As/As_*x*_O_*y*_@PDA@M NSs on two human cancer cell lines (A549 and MCF-7) and two human normal cell lines (HL-7702 liver cells and HEK293 embryonic kidney cells) were evaluated. As/As_*x*_O_*y*_@PDA@M NSs clearly showed significant cell inhibition ability on the two cancer cell lines with an inhibition percentage of 50% at a concentration of 10 μg/mL; by contrast, only 20% cell inhibition was detected in the two human normal cell lines, indicating significant selective killing effect of As/As_*x*_O_y_@PDA@M NSs (Fig. 8a). As mentioned before, As/As_*x*_O_*y*_ NSs could catalyze Fenton-like reactions and bind to anti-oxidants and anti-oxidases containing sulfhydryl groups, leading to the reduced ability of cell clearance and antagonism to oxidation to increase the intracellular ROS concentration. This might be the main reason for the significant selective cytotoxicity of As/As_*x*_O_*y*_@PDA@M NSs. As shown in Fig. 8b and Supplementary Fig. 9, although there was a degree of cell death after treatment with As/As_*x*_O_*y*_@PDA@M NSs plus 808 nm laser irradiation (PTT), dose-dependent severe cell death was observed when cells were treated with As/As_*x*_O_*y*_@PDA@M NSs plus 660 nm light irradiation (PDT), which indicated that the ROS burst was the main cause for serious cell death. In addition, the As/As_*x*_O_*y*_@PDA@M NSs-treated cells upon 660 and 808 nm laser irradiation showed significantly reduced viability, demonstrating the highly enhanced therapeutic effect by the ROS burst and PTT. To explore the mechanisms underlying these therapeutic outcomes, an apoptotic study was carried out. As detected by flow cytometry (Fig. 8c and Supplementary Fig. 10), early and late apoptotic cells with a percentage of 15.5% and 16.4%, respectively, were observed. As one of the earliest phenomena in cell apoptosis, a change in mitochondrial transmembrane potential was observed, confirming that apoptosis began after As/As_*x*_O_*y*_@PDA@M NSs treatment. JC-1 dye (5,5′,6,6′-tetrachloro-1,1′,3,3′-tetraethylbenzimidazolcarbocyanine iodide) was used to measure mitochondrial membrane potential (MMP) change. As presented in Fig. 8d, the depolarization of the mitochondria (an early apoptosis signal) was observed, further confirming the apoptosis of cancer cells induced by As/As_*x*_O_*y*_@PDA@M NSs. Moreover, as confirmed previously, the prepared type II heterojunction-functionalized As/As_*x*_O_*y*_@PDA@M NSs possess robust ability to generate ROS (e.g., ·O_2_^−^ and ^1^O_2_) and consume GSH under 660 nm laser irradiation, which would mediate a drastic ROS burst in cells to induce apoptosis of cancer cells. The numbers of dead and live cells in the different treatment groups further supported the effective therapeutic effect of As/As_*x*_O_*y*_@PDA@M NSs (Fig. 8e).

### Fluorescence imaging and antitumor strategy in vivo

**Fig. 9:**
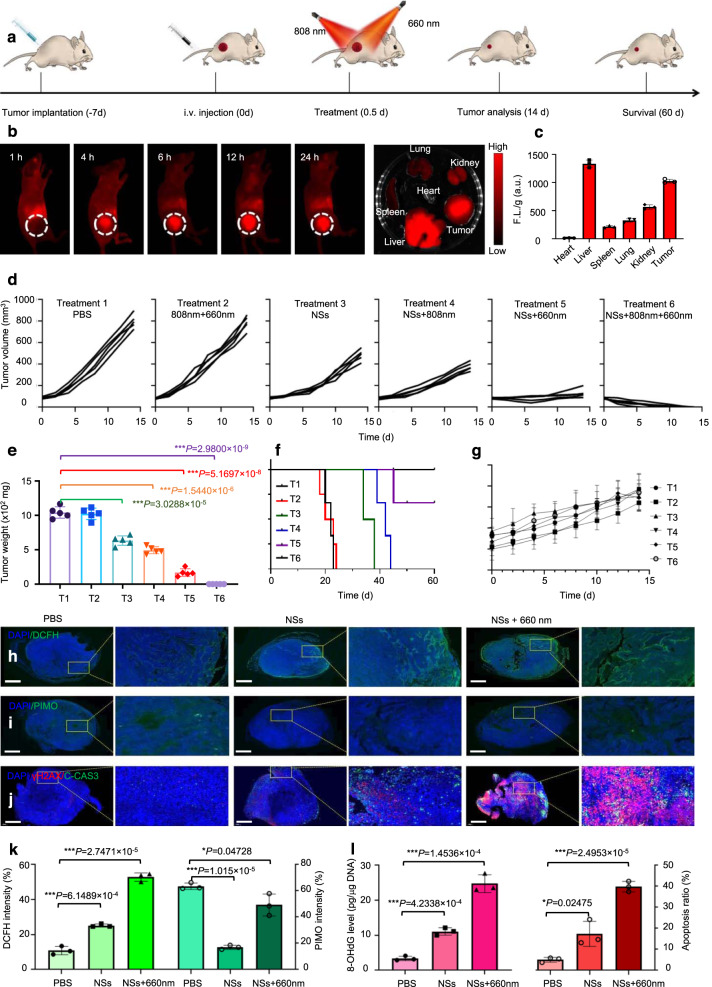
In vivo imaging and anti-tumor performance of As/As_*x*_O_*y*_@PDA@M NSs. **a** Treatment schedule. **b** In vivo fluorescence images of nude mice after i.v. administration of NSs, and the ex vivo fluorescence images of the tumor and major organs at 24 h post-injection of As/As_*x*_O_*y*_@PDA@M NSs with the excitation wavelength at 500 nm and emission wavelength at 795 nm. **c** Semiquantitative biodistribution of As/As_*x*_O_*y*_@PDA@M NSs in tumor and major organs 24 h post-injection. Error bars = standard deviation (*n* = 3), *n* = 3 biologically independent samples. **d** Tumor growth curves of MCF-7 tumor-bearing nude mice. **e** Tumor weight in different groups after 14 days of treatment. Error bars = standard deviation (*n* = 5), *n* = 5 biologically independent mice. **f** Survival rate of mice undergoing different treatments. **g** Body weight of mice during treatment. Error bars = standard deviation (*n* = 5), *n* = 5 biologically independent mice. **h** In vivo ROS detection in the sections from tumors by dichlorodihydrofluorescein (DCFH) via fluorescence microscopy, scale bars = 1000 μm. **i** In vivo O_2_ generation in sections from tumors by pimonidazole (PIMO) via fluorescence microscopy, scale bars = 1000 μm. **j** Immunofluorescence (IF) staining in tumor sections after treatment with PBS, NSs, or NSs + 660 nm laser irradiation, scale bars = 1000 μm. The nucleus is stained by DAPI (blue), damaged DNA by γH2AX foci (red), and apoptotic cells by apoptosis marker C-CAS3 (green). **k** Quantification of the in vivo ROS and O_2_ signals from the tumors calculated from the section studies in **h** and **i**. **l** In vivo DNA damage of tumors measured by 8-OHdG assay and apoptosis ratio after different treatments. Error bars = standard deviation (*n* = 3), *n* = 3 biologically independent samples. For all statistical analysis, data are presented as mean values ± SEM. Two-sided ANOVAs were performed for all other comparisons. No adjustments were made for multiple comparisons. For the in vivo fluorescence images and tumor IF staining, three times each experiment was repeated independently with similar results.

Encouraged by the excellent in vitro ROS burst ability, selective killing effect, as well as TEM-responsive degradation, the in vivo therapeutic outcomes of As/As_*x*_O_*y*_@PDA@M NSs were further investigated (Fig. 9a). To start with, owing to their intrinsic fluorescence properties, the in vivo fluorescence imaging ability of As/As_*x*_O_*y*_@PDA@M NSs were performed on the MCF-7 xenograft tumor model (via intravenous injection) to evaluate the biodistribution and tumor accumulation ability. As shown in Fig. 9b, the As/As_*x*_O_*y*_@PDA@M NSs effectively and continuously accumulated at the tumor site. At 24 h post injection, the mice were sacrificed and the tumors were dissected. As exhibited in Fig. 9b, the tumor showed bright fluorescence signal, which was consistent with the in vivo biodistribution results. Additionally, pharmacokinetics analysis (Supplementary Fig. 11) demonstrated the long circulation time of As/As_*x*_O_*y*_@PDA@M NSs. Although the As/As_*x*_O_*y*_@PDA@M NSs also accumulated in some organs, such as the liver, spleen, and lung, their relatively good biocompatibility and low dosage guarantee negligible damage to these organs.

Bearing an excellent tumor accumulation ability, the in vivo therapeutic studies of As/As_*x*_O_*y*_@PDA@M NSs were carried out. MCF-7 tumor-bearing mice were randomly separated into six groups and received different treatments: Group 1: saline; Group 2: 660 nm + 808 nm lasers; Group 3: As/As_*x*_O_*y*_@PDA@M NSs; Group 4: As/As_x_O_y_@PDA@M NSs + 808 nm laser; Group 5: As/As_x_O_y_@PDA@M NSs + 660 nm laser; and Group 6: As/As_x_O_y_@PDA@M NSs + 660 nm + 808 nm lasers. As/As_x_O_y_@PDA@M NSs (3 mg kg^−1^) were intravenously injected into mice in Groups 3–6. Both laser treatments (660 nm at 0.3 W cm^−2^ for 10 min and 808 nm at 1.0 W cm^−2^ for 10 min) were carried out at 24 h post injection of As/As_*x*_O_*y*_@PDA@M NSs. As revealed in Supplementary Figs. 12 and 13, significant temperature increases were observed in mice received As/As_*x*_O_*y*_@PDA@M NSs + 808 nm laser (Group 4) or As/As_*x*_O_*y*_@PDA@M NSs + 660 nm + 808 nm lasers (Group 6); by contrast no obvious changes were observed in mice that received only laser treatment, indicating the high photothermal conversion ability of As/As_*x*_O_*y*_@PDA@M NSs in vivo. Then a course of therapeutic treatment was carried out. No significant inhibition of tumor growth was observed in the control (Group 1) or laser-only (Group 2) groups, but obvious inhibition of tumor growth was observed in As/As_*x*_O_*y*_@PDA@M NSs-treated mice (Group 3). We attributed this to the accumulation of ROS through mediating Fenton-like reaction and inactivating some main anti-oxidants and anti-oxidases containing sulfhydryl groups to induce cancer cell apoptosis. Better therapeutic effects were observed in Group 4 (PTT) compared to Group 2, indicating the advantages of As/As_*x*_O_*y*_@PDA@M NSs as photothermal agents. Notably, a high level of inhibited tumor growth was observed in Group 5 because of the As/As_*x*_O_*y*_@PDA@M-based nanomedicine with multiple independently targeted ROS burst. After 4 days, portions of the tumors recrudesced but exhibited a relatively slow growth rate compared with that of the control group. The highly effective therapeutic effect of the combined As/As_*x*_O_*y*_@PDA@M NSs and 660 nm laser irradiation was attributed to the dramatic ROS burst achieved via tripartite ROS generation and the blocked two main ROS consumption pathways. Of note, nearly complete elimination of tumors without recurrence was achieved in mice treated with As/As_*x*_O_*y*_@PDA@M NSs and 660 or 808 nm laser irradiation (Group 6), indicating the remarkably enhanced therapeutic effect of combined PTT and ROS burst. The weight of excised tumors from euthanized mice (Fig. 9e) showed further evidence for the excellent therapeutic outcomes of PTT combined with ROS burst. Correspondingly, As/As_*x*_O_*y*_@PDA@M coupled with 660 and 808 nm laser treatment was associated with a high survival rate (Fig. 9f). More importantly, no notable side effects were observed in the above treatment groups (Fig. 9g).

The ROS burst effect was also studied by staining with a DCFH-DA. The results in Fig. 9h show a slight ROS accumulation after treatment with As/As_*x*_O_*y*_@PDA@M NSs, which was ascribed to mediation of a Fenton-like reaction and inactivation of some main anti-oxidants and anti-oxidases. Moreover, strong green fluorescence was detected in the As/As_*x*_O_*y*_@PDA@M NSs + 660 nm laser group, further indicating a drastic ROS burst in tumor cells. The Fenton-like reaction ability of As/As_*x*_O_*y*_@PDA@M NSs, such as the generation of O_2_, was also confirmed by the remission of hypoxic microenvironment exhibited in Fig. 9i. As previously reported^46^, the reason for ROS-induced cell toxicity is mainly DNA damage caused by ROS. We thus further assessed DNA damage levels and cell apoptosis in tumors after different treatment using γ-H2AX and C-CAS3 as markers for DNA double-strand breaks and cell apoptosis, respectively^47^. Treatment with As/As_*x*_O_*y*_@PDA@M NSs alone induced a certain extent of irreparable DNA damage and cancer cell apoptosis. When 660 nm laser irradiation was added, remarkably high levels of irreparable DNA damage and cell apoptosis in tumor section were observed (Fig. 9j). To further demonstrate this potential mechanism, 8-hydroxy-2′-deoxyguanosine (8-OHdG)—a reliable marker for oxidative stress—was detected after different treatments (Fig. 9k). As displayed in Fig. 9l, the 8-OHdG results showed trends consistent with the γ-H2AX data. Collectively, these results further confirmed that the therapeutic strategy based on As/As_*x*_O_*y*_@PDA@M NSs could efficiently induce a ROS burst and specifically kill effect on cancer cells.

### Biocompatibility evaluation

**Fig. 10:**
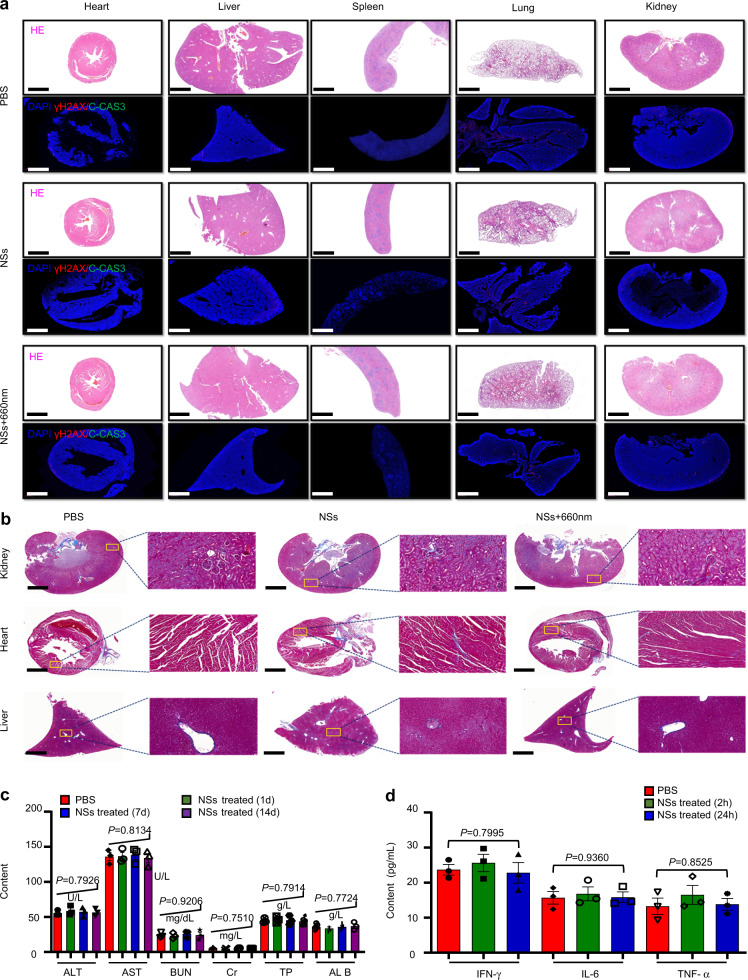
Biocompatibility evaluation of As/As_*x*_O_*y*_@PDA@M NSs. **a** H&E staining and immunofluorescence (IF) staining in sections from major organs after different treatments with PBS, NSs, or NSs + 660 nm laser irradiation (irradiation performed only within tumor areas). The nucleus was stained by DAPI (blue), damaged DNA by γH2AX foci (red), and apoptotic cells by apoptosis marker C-CAS3 (green). Scale bars = 1000 μm. **b** Masson staining of pathological fibrous deposition in main organs (kidney, heart, and liver) from mice treated with PBS, NSs, or NSs + 660 nm laser irradiation (irradiations performed only within tumor areas). Scale bars = 1000 μm. **c** Blood biochemistry and hematology analysis of Balb/c mice treated with As/As_*x*_O_*y*_@PDA@M NSs. **d** Serum levels IFN-γ, IL-6, and TNF-α in healthy mice at 2 or 24 h post intravenous injection of PBS or As/As_*x*_O_*y*_@PDA@M NSs. Error bars = standard deviation (*n* = 3), *n* = 3 biologically independent mice. For all statistical analysis, data are presented as mean values ± SEM. Two-sided ANOVAs were performed for all other comparisons. No adjustments were made for multiple comparisons. For the H&E staining, IF staining, and Masson staining, three times each experiment was repeated independently with similar results.

Considering that the in vivo toxicity of the materials plays an important role in the translation from bench to practical applications, we further set forth to evaluate the toxicity of As/As_*x*_O_*y*_@PDA@M NSs. We assessed DNA damage levels through γ-H2AX staining, apoptosis levels through cleaved caspase-3 (C-CAS3) staining, and performed histological analyses through H&E staining in vivo. As shown in Fig. 10a, no DNA damage, apoptosis, or signs of organ injury were detected in normal tissues, confirming the excellent in vivo biocompatibility of the As/As_*x*_O_*y*_@PDA@M NSs. Further, Masson staining of pathological fibrous deposition in main organs (kidney, heart, and liver) from mice treated with PBS, As/As_*x*_O_*y*_@PDA@M NSs, or As/As_*x*_O_*y*_@PDA@M NSs + 660 nm laser irradiation were performed. Sections from mice treated with NSs (with or without 660 nm laser irradiation) showed normal structures and no greater collagen deposition (blue staining) than sections from the PBS control group (Fig. 10b). Hence, these results clearly demonstrated that this therapeutic strategy based on As/As_*x*_O_*y*_@PDA@M NSs specifically targets tumor tissues in vivo while maintaining biocompatibility within normal tissues. Moreover, hematology assay, histology examination, and immune analysis further demonstrate biocompatibility. For the hematology and histology assay, blood examination was conducted at 1, 7, and 14 days post i.v. injection of As/As_*x*_O_*y*_@PDA@M NSs. As shown in Fig. 10c, no statistically significant differences in the amount of creatinine, aspartate aminotransferase (AST), albumin, total protein (TP), alanine aminotransferase (ALT), or blood urea nitrogen (BUN) were detected in mice that received As/As_*x*_O_*y*_@PDA@M NSs compared with that of control mice. For immune toxicity analysis, the amount of IFN-γ, IL-6, and TNF-α in serum samples from mice at 2 and 24 h post i.v. injection of As/As_*x*_O_*y*_@PDA@M NSs were measured. Figure 10d shows that all cytokine levels were nearly the same as those in the control group, further confirming that the obtained As/As_*x*_O_*y*_@PDA@M NSs are biocompatible in vivo.

## Discussions

During past years, ultrathin two-dimensional (2D) nanomaterials have received a great deal of attention for both fundamental studies and practical applications owing to their unique properties such as large surface-to-volume ratio, high charge-carrier mobility, tunable direct-bandgap, mechanical flexibility, etc. Among the 2D nanomaterials family, 2D monoelemental nanosheets (Xenes), such as graphene, BP, antimonene, borophene, arsenene, etc., have emerged as an unique family of nanomaterials that show numerous advantages and superior performances in biomedicine as a result of their unique physicochemical features^15,17–20,22^. Compared with other multielemental nanosheets (transition metal dichalcogenides, MXenes, etc.), Xenes with a simple elemental composition decrease the materials toxicity in biological system. The atomic-scale thickness makes Xenes invaluable for drug/gene delivery and stimuli-responsive systems that require extensive surface interactions on a small scale. The excellent optical and electrical properties enable Xenes as potential biological fluorescence and/or photoacoustic imaging agents for cancer diagnosis, and optical therapy platforms. For example, graphene, BP, antimonene, and borophene have been successfully used as photothermal agents and drug/gene delivery carriers for cancer PTT and chemo/gene therapy^18–20,22^. In addition, BP with semiconductor optical properties has been applied as an efficient photosensitizer for cancer PDT^48,49^. Arsenene, especially the as-fabricated As/As_*x*_O_*y*_ NSs in this study, exhibit excellent totipotency and pluripotency compared to the other Xenes. Firstly, As/As_*x*_O_*y*_ NSs with large surface-to-volume ratio and high photothermal conversion efficiency could be applied as drug/gene delivery carriers and photothermal agents for cancer chemo/gene therapy and PTT. Secondly, As/As_*x*_O_*y*_ NSs as type II heterojunction showed efficient ROS (^1^O_2_ and ·O_2_^−^) generation and GSH consumption, which mediated intracellular ROS burst and apoptosis of cancer cells. Thirdly, different from other Xenes, As/As_*x*_O_*y*_ NSs also possess ability to modulate TME and catalyze Fenton-like reaction to provide substrates of O_2_ for enhancing PDT efficacy, resulting in the increased intracellular ROS for accelerating apoptosis of cancer cells. Fourthly, As/As_*x*_O_*y*_ NSs exhibit unique anti-tumor property through inactivating some main anti-oxidants and anti-oxidases to cut off the “retreat routes” of ROS, and by damaging MMP. Last but not least, the inherent outstanding fluorescence imaging ability of As/As_*x*_O_*y*_ NSs endows them as in vivo imaging agents. In line of the remarkable biocompatibility and biodegradability, As/As_*x*_O_*y*_ NSs present good totipotency and versatility, endowing them with great potential in clinical application value and prospect.

In conclusion, our work presents a means of creating a nanomedicine based on 2D As/As_*x*_O_*y*_@PDA@M NSs capable of multiple independently targeted ROS bursts and a powerful capacity for tumor inhibition. The As_*x*_O_*y*_ indirectly induces the accumulation of intracellular ROS content by inactivating some main anti-oxidants and anti-oxidases to cut off the “retreat routes” of ROS. As acted as an efficient photosensitizer to catalyze the generation of ^1^O_2_ from O_2_ when exposed to the 660 nm laser, directly improving the intracellular ROS content. According to the band structure of As and As_*x*_O_*y*_, a promising Type II heterojunction was formed. Thus, the electrons in the CB of As preferred to transfer to the CB of As_*x*_O_*y*_. At the same time, the holes in the VB of As_*x*_O_*y*_ preferred to transfer to the VB of As, contributing to thoroughly separate photo-excited charges in the CB and VB of two different photosensitizers, further catalyzing the reduction of O_2_ to generate ·O_2_^−^ and oxidation of GSH to inhibit the antioxidant ability of cancer cells. As a consequence, the efficient photo-excited charge separation in the As/As_*x*_O_*y*_ NSs-based Type II heterojunction can enhance the intracellular ROS content in both direct and indirect ways. The abundant oxygen vacancies on the surface of As/As_*x*_O_*y*_ NSs can catalyze a Fenton-like reaction to generate ·OH and O_2_ from H_2_O_2_, which can not only directly promote ROS burst but also essentially enhance the PDT effect. Additionally, after surface engineering with PDA and M shells, the produced As/As_*x*_O_*y*_@PDA@M NSs not only possess remarkable biocompatibility with no long-term safety concerns in vivo, but also enhance the accumulation in tumors with homologous targeting ability to mediate high photothermal conversion efficiency under laser irradiation. The inherent outstanding fluorescence imaging ability of As/As_*x*_O_*y*_@PDA@M NSs further demonstrate the imaging-guided therapeutic performance both in vitro and in vivo. Therefore, such an unprecedented type II heterojunction successfully induces a ROS burst in cancer cells and integrates imaging-guided chemo/photothermal/photodynamic therapies, establishing a paradigm for cancer therapy with high efficacy therapeutic efficacy and high biocompatibility.

## Methods

### Materials

Bulk arsenic was purchased from Sigma-Aldrich and stored in the dark with argon protection. Human normal liver cells (HL-7702), human embryonic kidney 293 (HEK293), human non-small cell lung cancer (A549), and human breast cancer cell (MCF-7) were obtained from ATCC. DPBF, MB, H_2_O_2_ (30%), glutathione, and N-methyl-pyrrolidone (NMP) were purchased from Sigma-Aldrich. RPMI 1640 medium, Dulbecco’s modified Eagle medium (DMEM), PBS, fetal bovine serum (FBS) and trypsin-EDTA were purchased from Gibco Life Technologies. Primary antibodies: Phospho-Histone H2A.X (Ser139) (D7T2V), Cell Signaling (Product # 80312), Dilution 1:200; C-CAS3 (Asp175) (5A1E), Cell Signaling (Product # 9664), Dilution 1:250. Secondary antibodies: Anti-Rabbit IgG (H+L) Highly Cross-Adsorbed Secondary Antibody, Alexa Fluor 488, ThermoFisher (Catalog # A-11034), Dilution 1:1000; Anti-Mouse IgG (H+L) Highly Cross-Adsorbed Secondary Antibody, Alexa Fluor 647, ThermoFisher (Catalog # A-21236), Dilution 1:1000.

### Characterization

The morphology and thickness of NSs was assessed by transmission electron microscopy (TEM, JEM-2100UHR, JEOL, Japan) and atomic-force microscopy (AFM, FASTSCANBIO, Germany). Dynamic light scattering (DLS) was applied to study the size and zeta potential of NSs. XPS (ESCALAB 250Xi, Japan) and energy-dispersive X-ray spectroscopy (EDS) (Inca X-MAX, Oxford, UK) were used to detect NSs’s chemical constituents. The chemical structures were studied by Bruker D8 multipurpose XRD. The Infinite M200 PRO spectrophotometer was used to study the UV–Vis–NIR spectra. The electronic spin resonance (ESR) spectrum was employed to detect ROS radicals.

### Preparation of cancer cell membrane fragments

Cells were first incubated in culture dishes (10.0 cm in diameter), then detached with a cell scraper and followed by centrifugation at 700×*g* for 5 min. Cells were collected and resuspended in the cold PBS buffer, followed by centrifugation at 600×*g* for 5 min to obtain cell pellets. The cell pellets were incubated with a hypotonic lysing buffer consisting of phenylmethanesulfonyl fluoride (PMSF) (Beyotime Institute of Biotechnology) and membrane protein extraction reagent in an ice bath for 10–15 min. Using a freeze–thaw method, cells were then broken repeatedly at 700 × *g* for 10 min at 4 °C. The supernatant was further centrifuged at 14,000 × *g* for 30 min to collect the cell membrane fragments and lyophilized overnight. The final materials were stored at −80 °C and rehydrated in ultra-pure water or PBS buffer before use.

### Synthesis of As/As_*x*_O_*y*_@PDA@M NSs

As/As_*x*_O_*y*_ NSs were synthesized by ball-milling and probe sonication-assisted liquid exfoliation. First, 1 g of As was dispersed in 80 mL NMP and ground by ball milling at 800 rpm for 30 min. Then the mixture was sonicated for 10 h in NMP. Finally, the solution was centrifuged at 1006×*g* for 5 min to obtain As/As_*x*_O_*y*_ NSs, which were stored at 4 °C. For the PDA coating, the resulting As/As_x_O_y_ NSs were dispersed in 10 mL of Tris buffer (pH 8.5, 10 × 10^–3^ M) containing dopamine hydrochloride (10 mg), and the above mixture was rotated for 4 h at room temperature in darkness to obtain PDA-coated As/As_*x*_O_*y*_ NSs (As/As_*x*_O_*y*_@PDA NSs). For the cancer cell membrane coating, the As/As_*x*_O_*y*_@PDA NSs were added into 10 mL of PBS containing cancer cell membrane at room temperature. Sonication was applied for 1 h to obtain cancer cell membrane-coated As/As_*x*_O_*y*_@PDA NSs (As/As_*x*_O_*y*_@PDA@M NSs).

### Photothermal conversion and stability

1 mL of As/As_*x*_O_*y*_@PDA@M NSs solution at different concentrations (10, 50, and 100) were irradiated by an 808 nm laser with different power density (0.5, 1, and 2 W/cm^2^) for 5 min. The temperature of the As/As_*x*_O_*y*_@PDA@M NSs solution was recorded every 30 s by an IR thermal camera. The stability of As/As_*x*_O_*y*_ NSs and As/As_*x*_O_y_@PDA@M NSs was investigated by evaluating their absorption and photothermal performance. First, As/As_*x*_O_*y*_ NSs or As/As_*x*_O_*y*_@PDA@M NSs were dispersed in PBS (pH = 7.4) solution. Then, their absorption and photothermal performance were detected at predetermined time intervals to characterize the stability of As/As_*x*_O_*y*_ NSs and As/As_*x*_O_*y*_@PDA@M NSs.

### Biocompatibility and cytotoxicity

Normal cells (HL-7702 and HEK 293) and cancer cells (A549 and MCF-7) were seeded in 96-well plates (37 °C, 5% CO_2_) and cultured for 24 h. The culture medium was then replaced with new fresh culture medium containing As/As_*x*_O_*y*_ NSs at different concentrations (0.5, 1, 5, and 10 μg/mL) and incubated for another 24 h. 3-(4,5-dimethylthiazol-2-yl)−2,5-diphenyltetrazolium bromide (MTT) assay was used to determine cell viability.

### Cellular uptake

The uptake efficacy of As/As_*x*_O_*y*_ NSs, As/As_*x*_O_*y*_@PDA NSs, and As/As_*x*_O_*y*_@PDA@M NSs was studied. 5 × 10^4^ MCF-7 cells were incubated in glass-bottomed dishes (1.5 mL of cell culture medium) for 12 h (37 °C, 5% CO_2_). Then the As/As_*x*_O_*y*_ NSs, As/As_*x*_O_*y*_@PDA NSs, or As/As_*x*_O_*y*_@PDA@M NSs were added with an As dose of 5 µg/mL. After incubation for 4 h, cells were rinsed carefully with PBS twice and fixed by paraformaldehyde (4%) for 15 min. Then the cells were washed with PBS twice and incubated with 500 µL of Hoechst 33342 fluorescent dye for 30 min. Finally, confocal laser scanning microscopy (CLSM) was applied for fluorescent imaging. Hoechst 33342 was excited at 350 nm, while As/As_*x*_O_y_ NSs were excited at 400 nm.

### ROS production and GSH consumption in vitro

1, 3-diphenylisobenzofuran (DPBF) was applied to assess the ability of As/As_*x*_O_*y*_@PDA@M NSs to generate ·O_2_^−^ and ^1^O_2_. In brief, As/As_*x*_O_*y*_@PDA@M NSs were mixed with DPBF (1 mL, 1 mM) at a final volume of 3 mL. The final concentrations of As/As_*x*_O_*y*_@PDA@M NSs were 5, 10, and 15 μg/mL. The mixture was agitated in dark for 1 h to reach a balance of desorption and adsorption. Then a 660 nm laser (0.3 W/cm^2^) was applied to irradiate the reaction solution. The concentration of DPBF was measured by the UV–Vis spectrophotometer. A GSH assay kit was employed to measure the consumption of GSH under the same conditions. The ·OH generation of As/As_*x*_O_*y*_@PDA@M NSs was determined by MB. Briefly, As/As_*x*_O_*y*_@PDA@M NSs suspension was mixed with MB and H_2_O_2_ in PBS, with final concentrations of As/As_*x*_O_*y*_@PDA@M NSs being 5, 10, or 15 μg/mL. The absorbance of MB was recorded by UV–vis–NIR after different time intervals.

### ROS production in cells

The ROS production was detected by DCFH-DA. Briefly, MCF-7 cells were cultured in dishes for 24 h (37 °C, 5% CO_2_). Then, fresh medium containing As/As_*x*_O_*y*_@PDA@M NSs was added to replace the original medium, and cells were allowed to culture for another 12 h. After that, the DCFH-DA solution (0.2 mM) was added and cells were cultured for 0.5 h and washed by PBS three times. Finally, cells were exposed to a 660 nm laser (0.3 W/cm^2^) for 10 min, with green fluorescence imaged by CLSM indicating the generation of ROS.

### In vitro therapy with As/As_*x*_O_*y*_@PDA@M NSs

A549 and MCF-7 cells were seeded and cultured in 96-well plates (37 °C, 5% CO_2_). After 24 h, medium was discarded and fresh medium containing As/As_*x*_O_*y*_@PDA@M NSs (0.5, 1, 5, and 10 μg/mL) was added for another 12 h incubation. Then cells were washed with PBS and irradiated by a 660 nm laser (0.3 W/cm^2^) or an 808 nm laser (1.0 W/cm^2^) in fresh medium for 10 min. After incubation for 12 h, PBS was used to wash the cells three times, and the MTT assay was used to assess cell viability.

### Animals

All animals received humane care, and the Animal Ethics Committee of the National Institute of Radiological Sciences approved all the animal experiments. All experiments were carried out according to the recommendations of the Committee for the Care and Use of Laboratory Animals, Tianjin University. The cages were placed in conventional rooms with controlled photoperiod (07:00–19:00 h white light, ±200 lx at 1 m above the floor; 19:00–07:00 h red light, ±5 lx at 1 m), temperature (20–22 °C), relative humidity (50–60%), and ventilation (15 air changes h^−1^).

### Pharmacokinetic study

C57BL/6 mice were administered As/As_*x*_O_*y*_@PDA@M NSs (200 μL, 2 mg/kg) via intravenous (i.v.) injection. 20 mL blood sample was collected to study the fluorescence of As/As_*x*_O_*y*_ NSs at various lengths.

### Xenograft tumor model

To build the xenograft tumor model, 200 μL MCF-7 cells (2 × 10^6^ cells) were subcutaneously injected into naked Balb/c mice to obtain a tumor volume of ~100 mm^3^ (calculated as tumor length × tumor width × tumor width/2) for the following experiments.

### In vivo biodistribution and fluorescence imaging study

As/As_*x*_O_*y*_@PDA@M NSs were i.v. injected into tumor-bearing mice, and the fluorescence was measured by the Maestro2 In-Vivo Imaging System. At 24 h post-injection, tumor and major organs were collected and the concentration of As/As_*x*_O_*y*_@PDA@M NSs accumulated in tumor and major organs was quantified by the fluorescence intensity.

### In vivo therapy of As/As_*x*_O_*y*_@PDA@M NSs

Thirty tumor-bearing mice were randomly divided into six groups for different treatments^50,51^. G1: saline as control group, G2: 808 and 660 nm laser irradiation, G3: As/As_*x*_O_*y*_@PDA@M NSs (2 mg/kg), G4: As/As_*x*_O_*y*_@PDA@M NSs with 808 nm laser (1.0 W/cm^2^), G5: As/As_*x*_O_*y*_@PDA@M NSs with 660 nm laser (0.3 W/cm^2^), and G6: As/As_*x*_O_*y*_@PDA@M NSs with 660 and 808 nm laser irradiation. The tumor volume was recorded every two days during the course of study of 14 days.

### In vivo toxicity

C57BL/6 mice were i.v. injected with As/As_*x*_O_*y*_@PDA@M NSs (5 mg/kg). Blood samples were collected to measure interleukin6 (IL-6), tumor necrosis factor-α (TNF-α), and interferon-γ (IFN-γ) by ELISA at 12 and 24 h post-injection. At days 1, 7, and 14, albumin (ALB), aspartate ALT, AST, BUN, creatinine (Cr), and TP were measured via a complete blood panel test. For in vivo toxicity, mice were sacrificed to obtain their main organs for eosin (H&E) and hematoxylin staining after 30 days.

### Statistical analysis

Analysis of flow cytometry data was performed with Flowjo v7.6 software. Graph Pad Prism (8.0) and Origin 9.0 were used for data statistics and statistical significance calculation. Microsoft Excel 2016 was used for biodistribution and tumor size analysis. FL images were analyzed using Image J 1.8.0. Zetasizer Nano software v3.30 for analyses of particle size. TEM data was analyzed using Gatan-DigitalMicrograph-3.9. AFM data was analyzed using NanoScope Analysis 1.8. Statistical analysis was performed using the Student’s *t*-test with statistical significance assigned at *P < 0.05 (significant), **P < 0.01 (moderately significant), ***P < 0.001 (highly significant), and ****P < 0.0001 (extremely significant).

### Reporting summary

Further information on research design is available in the Nature Research Reporting Summary linked to this article.

## Supplementary information

Supplementary Information

Reporting Summary

## Data Availability

The authors declare that all data supporting the findings of this study are available within the article and the Supplementary Information. Other data are available from the corresponding authors upon reasonable request.
