# Supplementary material for: Arsenene-mediated multiple independently targeted reactive oxygen species burst for cancer therapy
Source: Nat Commun. 2021 Aug 6;12:4777. doi: 10.1038/s41467-021-24961-5 (PMC8346549; doi:10.1038/s41467-021-24961-5)
Supplement: Supplementary file 1 — Supplementary Information [file 41467_2021_24961_MOESM1_ESM.pdf]

Supplementary Information

Arsenene-mediated multiple independently targeted reactive oxygen species burst for cancer therapy

Na Kong,[1] Hanjie Zhang,[2] Chan Feng,[1] Chuang Liu,[1] Yufen Xiao,[1] Xingcai Zhang,[3] Lin Mei,[4] Jong Seung Kim,[5] Wei Tao,[1*] Xiaoyuan Ji[1,2*]

[1] Center for Nanomedicine, Brigham and Women's Hospital, Harvard Medical School, Boston, MA 02115, USA.

[2]Academy of Medical Engineering and Translational Medicine, Medical College, Tianjin University, Tianjin 300072, China.

[3] School of Engineering and Applied Sciences, Harvard University, Cambridge, MA, USA.

[4]Tianjin Key Laboratory of Biomedical Materials, Key Laboratory of Biomaterials and Nanotechnology for Cancer Immunotherapy, Institute of Biomedical Engineering, Chinese Academy of Medical Sciences and Peking Union Medical College, Tianjin 300192, China.

[5] Department of Chemistry, Korea University, Seoul, 02841, Korea.

[*]Corresponding author.

Email: wtao@bwh.harvard.edu (W. Tao); jixiaoyuan@tju.edu.cn (X. Ji)

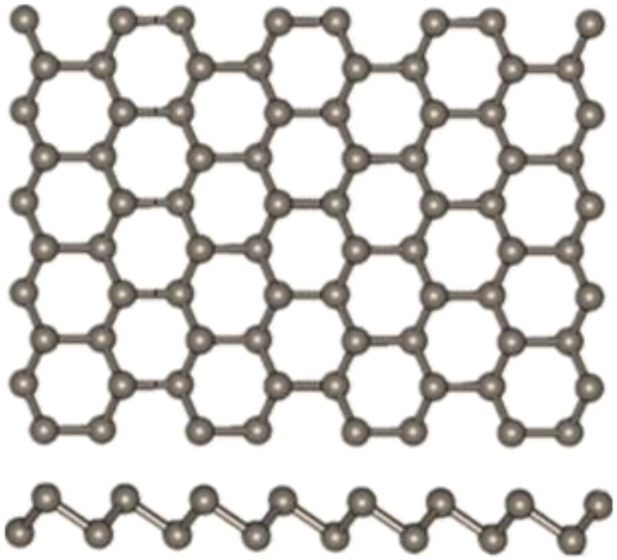

Supplementary Fig. 1 Top view and side view of geometrical structures of monolayer arsenene.

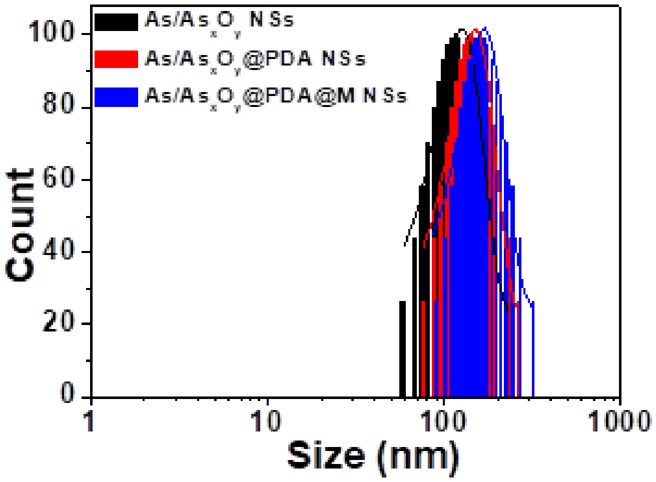

Supplementary Fig. 2 Size distribution of As/As$_x$O$_y$ NSs, As/As$_x$O$_y$@PDA NSs, and As/As$_x$O$_y$@PDA@M NSs.

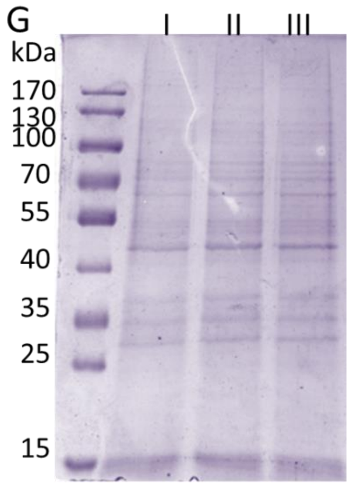

Supplementary Fig. 3 SDS-PAGE protein analysis using Coomassie blue staining. I: Cancer cell lysate, II: cancer cell membrane vesicles, III: As/As_xO_y@PDA@M NSs. Three times of this experiment was repeated independently with similar results.

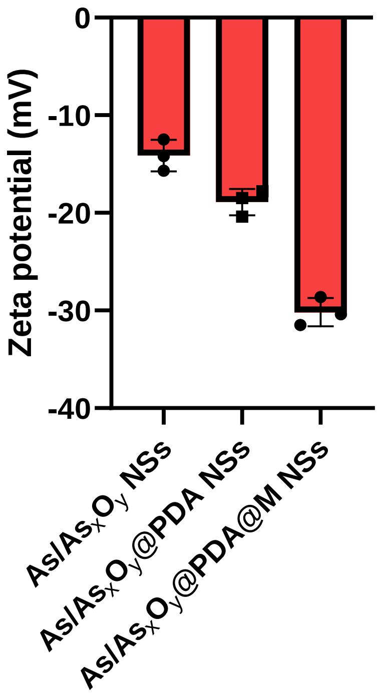

Supplementary Fig. 4 Zeta potential of As/As_xO_y NSs, As/As_xO_y@PDA NSs, and As/As_xO_y@PDA@M NSs. Error bars = Standard Deviation (n=3), n=3 biologically independent samples. Data are presented as mean values +/- SEM. Two-sided ANOVAs were performed for all other comparisons. No adjustments were made for multiple comparisons.

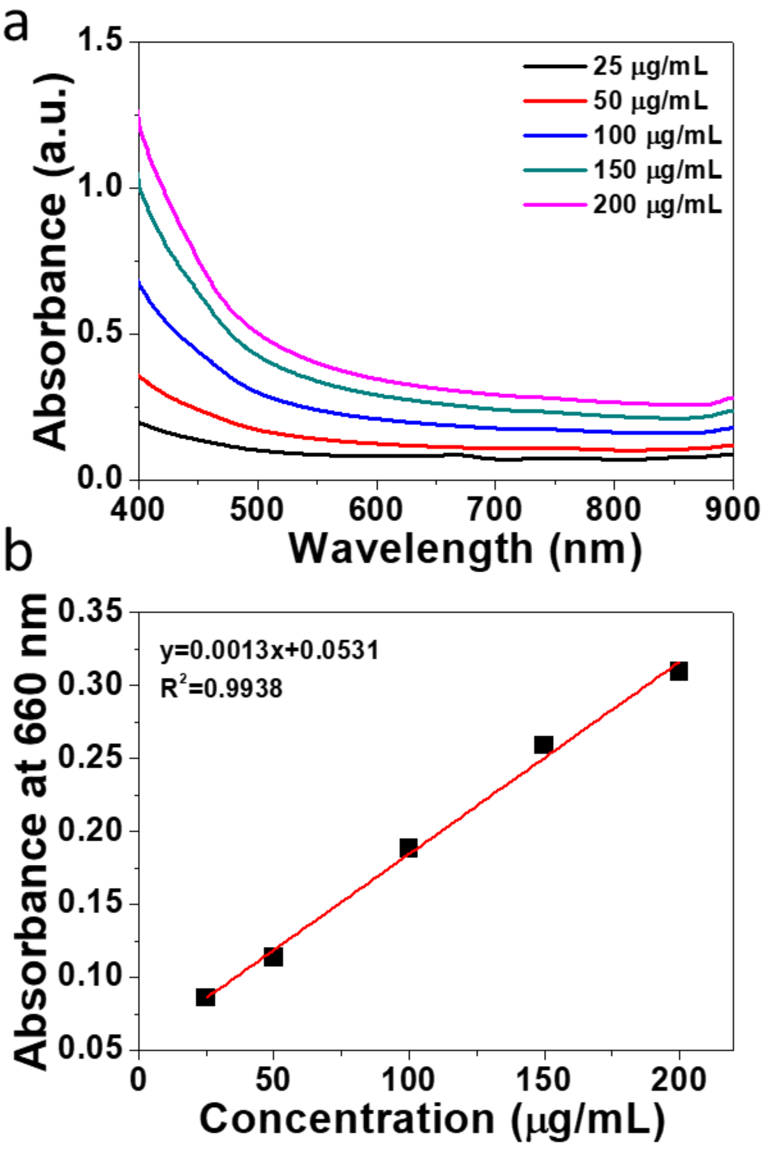

Supplementary Fig. 5 a UV-Vis absorbance spectra of As/As_xO_y@PDA@M NSs with different concentrations in water. **b** Normalized absorbance intensity of As/As_xO_y@PDA@M NSs divided by the characteristic length of the cell (A/L) at different concentrations for λ=808 nm.

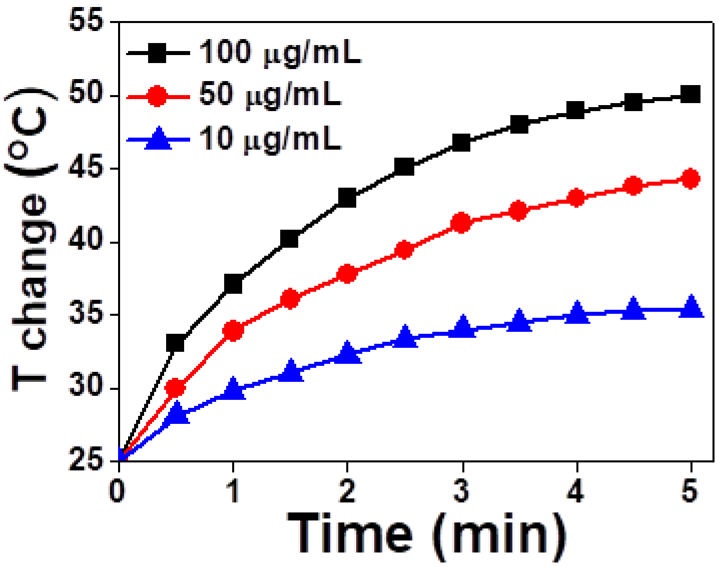

Supplementary Fig. 6 Photothermal conversion curves of As/As_xO_y@PDA@M NSs solution.

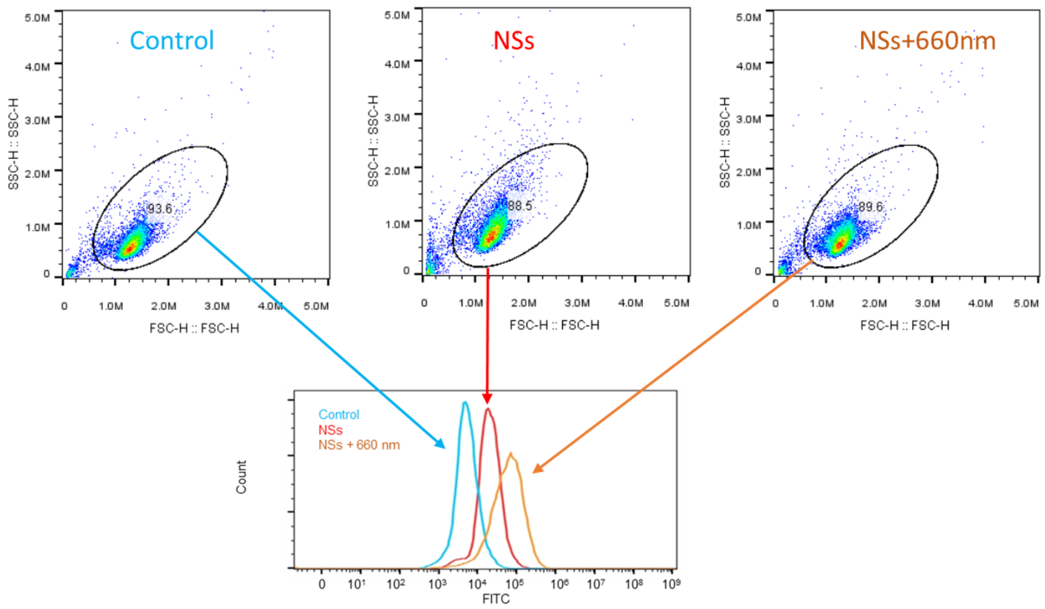

Supplementary Fig. 7 Gating strategy to determine the intracellular ROS content after different treatments. Three times of this experiment was repeated independently with similar results.

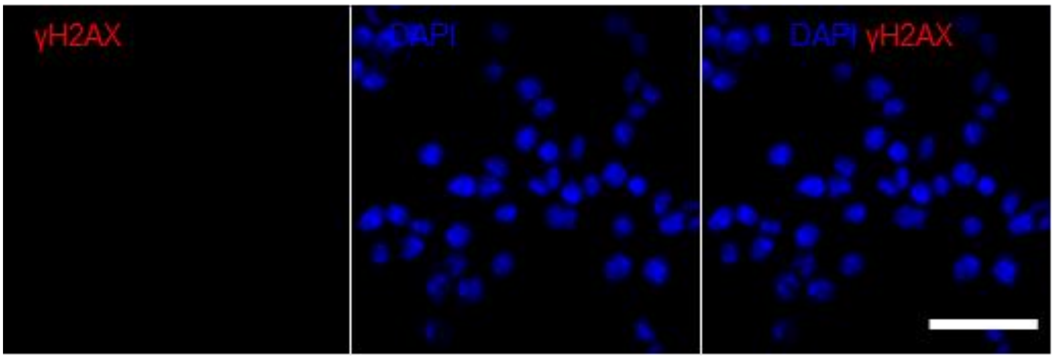

Supplementary Fig. 8 Representative confocal microscopy images of the MCF-7 cells (scale bars, 50 μm) without any treatment. The nuclei were stained by DAPI (blue), and the γH2AX foci per nucleus were stained by anti-γH2AX antibody (red). Three times of this experiment was repeated independently with similar results.

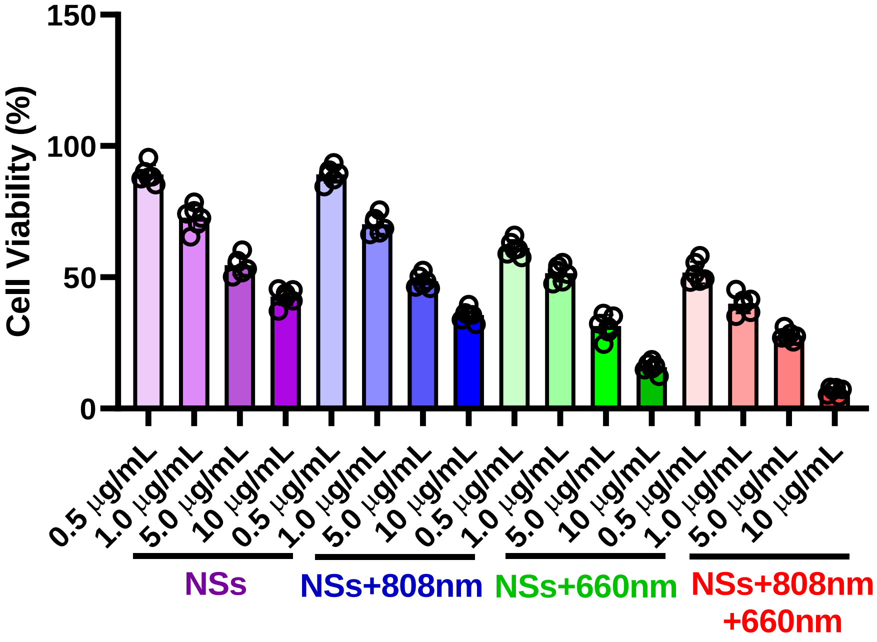

Supplementary Fig. 9 Antitumor efficacy of As/As_xO_y@PDA@M NSs at different concentrations on A549 cells. The power of 660 nm laser and 808 nm laser were 0.3 and 1.0 W/cm^2 ,respectively. The exposure time was 10 min. Error bars = Standard Deviation (n=6), n=6 biologically independent cells. Data are presented as mean values +/- SEM. Two-sided ANOVAs were performed for all other comparisons. No adjustments were made for multiple comparisons.

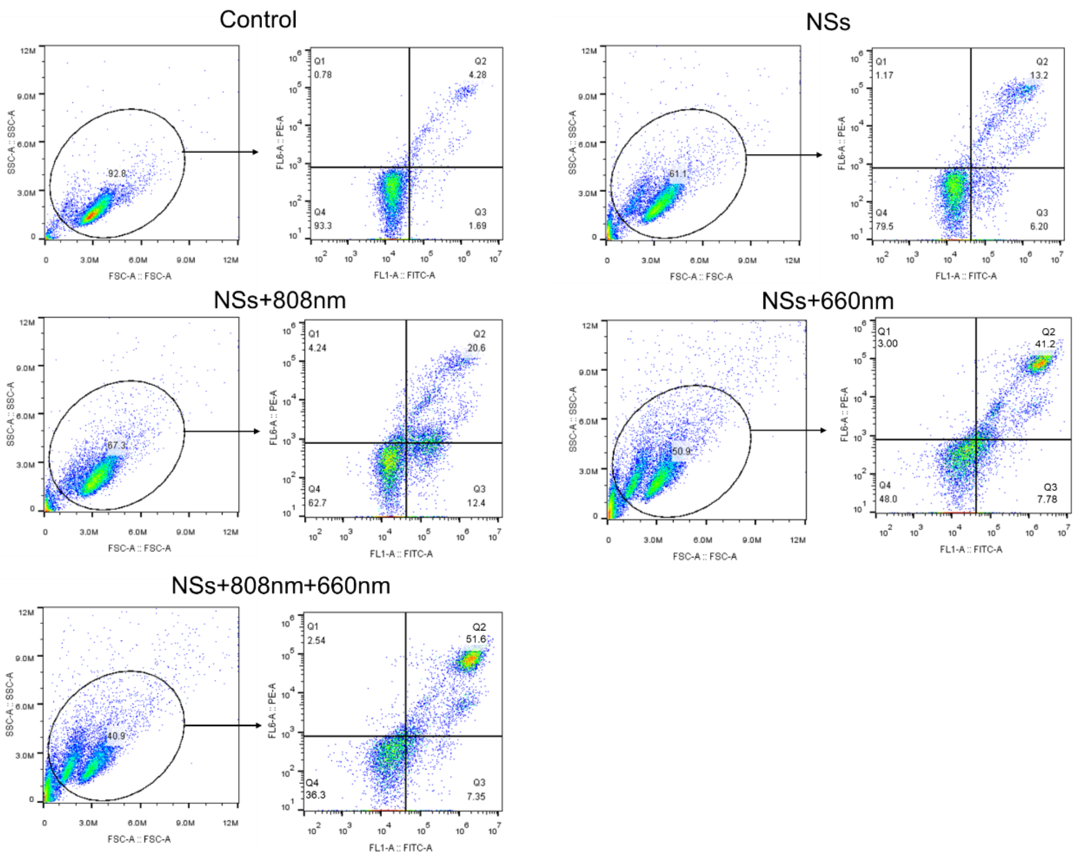

Supplementary Fig. 10 Gating strategy to determine the percentage of cell apoptosis after different treatments. Three times of this experiment was repeated independently with similar results.

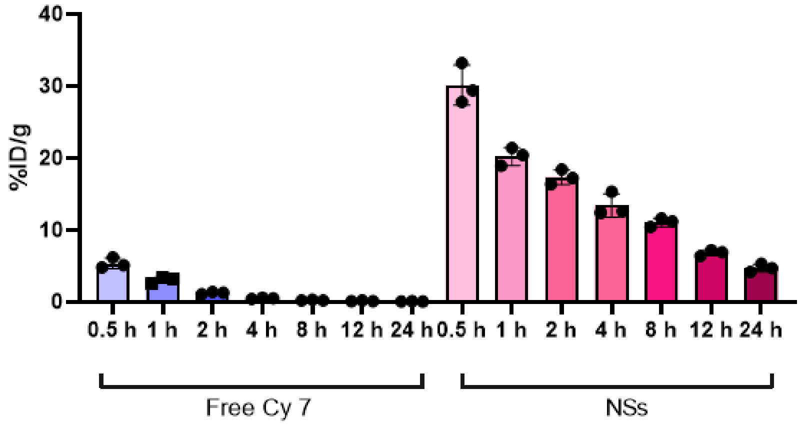

Supplementary Fig. 11 Blood circulation of As/As_xO_y@PDA@M NSs. Error bars = Standard Deviation (n=3), n=3 biologically independent mice. Data are presented as mean values +/- SEM. Two-sided ANOVAs were performed for all other comparisons. No adjustments were made for multiple comparisons.

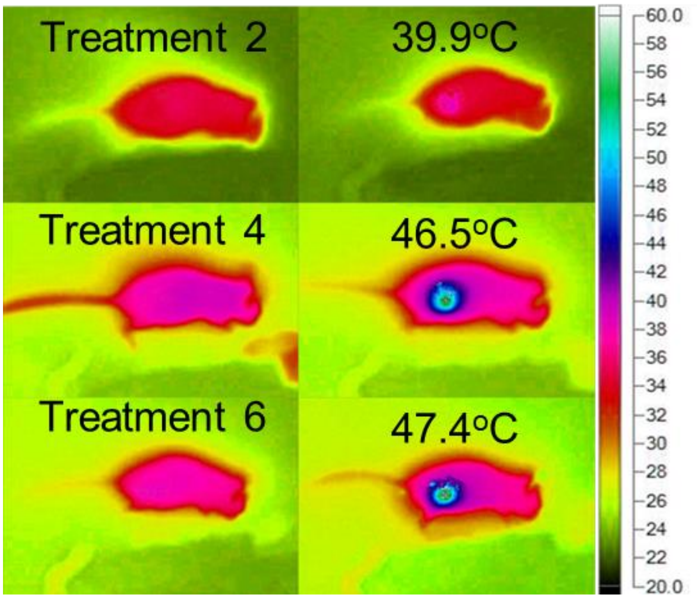

Supplementary Fig. 12 Infrared thermographic images of MCF-7 tumor-bearing nude mice under different treatments. The power of 660 nm laser and 808 nm laser were 0.3 and 1.0 W/cm^2, respectively. The exposure time was 10 min.

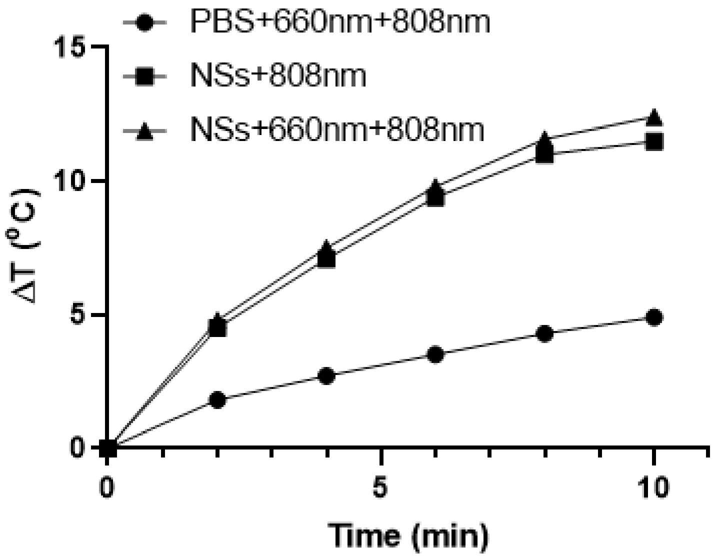

Supplementary Fig. 13 Time-dependent temperature changes in MCF-7 tumor-bearing nude mice after different treatments.
